# Cost‐effectiveness analysis of potentially curative and combination treatments for hepatocellular carcinoma with person‐level data in a Canadian setting

**DOI:** 10.1002/cam4.1119

**Published:** 2017-08-08

**Authors:** Hla‐Hla Thein, Wanrudee Isaranuwatchai, Yao Qiao, Kenny Wong, Gonzalo Sapisochin, Kelvin K. W. Chan, Eric M. Yoshida, Craig C. Earle

**Affiliations:** ^1^ Dalla Lana School of Public Health University of Toronto Toronto Ontario Canada; ^2^ Institute for Clinical Evaluative Sciences Toronto Ontario Canada; ^3^ Centre for Excellence in Economic Analysis Research The HUB Li Ka Shing Knowledge Institute St. Michael's Hospital Toronto Ontario Canada; ^4^ Institute of Health Policy Management and Evaluation University of Toronto Toronto Ontario Canada; ^5^ Multi‐Organ Transplant Division of General Surgery University Health Network University of Toronto Toronto Ontario Canada; ^6^ Odette Cancer Centre Sunnybrook Health Sciences Centre Toronto Ontario Canada; ^7^ Department of Medicine University of Toronto Toronto Ontario Canada; ^8^ Canadian Centre for Applied Research in Cancer Control (ARCC) Toronto Ontario Canada; ^9^ University of British Columbia Division of Gastroenterology Vancouver British Columbia Canada; ^10^ Ontario Institute for Cancer Research Toronto Ontario Canada; ^11^ Cancer Care Ontario Toronto Ontario Canada

**Keywords:** Cost, cost‐effectiveness acceptability curve, economic evaluation, effect, intervention, liver cancer

## Abstract

Patients with early‐stage hepatocellular carcinoma (HCC) are potential candidates for curative treatments such as radiofrequency ablation (RFA), surgical resection (SR), or liver transplantation (LT), which have demonstrated a significant survival benefit. We aimed to estimate the cost‐effectiveness of curative and combination treatment strategies among patients diagnosed with HCC during 2002–2010. This study used Ontario Cancer Registry‐linked administrative data to estimate effectiveness and costs (2013 USD) of the treatment strategies from the healthcare payer's perspective. Multiple imputation by logistic regression was used to handle missing data. A net benefit regression approach of baseline important covariates and propensity score adjustment were used to calculate incremental net benefit to generate incremental cost‐effectiveness ratio (ICER) and uncertainty measures. Among 2,222 patients diagnosed with HCC, 10.5%, 14.1%, and 10.3% received RFA, SR, and LT monotherapy, respectively; 0.5–3.1% dual treatments; and 0.5% triple treatments. Compared with no treatment (53.2%), transarterial chemoembolization (TACE) + RFA (average $2,465, 95% CI: −$20,000–$36,600/quality‐adjusted life years [QALY]) or RFA monotherapy ($15,553, 95% CI: $3,500–$28,500/QALY) appears to be the most cost‐effective modality with lowest ICER value. The cost‐effectiveness acceptability curve showed that if the relevant threshold was $50,000/QALY, RFA monotherapy and TACE+ RFA would have a cost‐effectiveness probability of 100%. Strategies using LT delivered the most additional QALYs and became cost‐effective at a threshold of $77,000/QALY. Our findings found that TACE+ RFA dual treatment or RFA monotherapy appears to be the most cost‐effective curative treatment for patients with potential early stage of HCC in Ontario. These findings highlight the importance of identifying and measuring differential benefits, costs, and cost‐effectiveness of alternative HCC curative treatments in order to evaluate whether they are providing good value for money in the real world.

## Introduction

Liver cancer is one of the few cancers that is increasing in incidence and mortality worldwide 1, including Canada 2, 3, 4. This is due to the growing prevalence of underlying chronic liver diseases, mainly chronic viral hepatitis, alcoholic and nonalcoholic liver disease, and the aging of the population that have those diseases 2, 3, 4. Hepatocellular carcinoma (HCC) accounts for the majority (~72%) of liver cancers in both males and females in Canada 4. For patients with early‐stage HCC, potentially curative treatment options include radiofrequency ablation (RFA), surgical resection (SR), and liver transplantation (LT). These treatments provide survival benefits, and outcomes are optimized by identification of appropriate patients 5, 6, 7. Transarterial chemoembolization (TACE) is the standard of care for patients with intermediate‐stage HCC 8, 9, 10. However, in clinical practice, TACE has been used as an alternative or combination therapy in patients with early‐ or advanced‐stage HCC 11.

HCC is associated with high costs for treatment, in particular, SR and LT, and management of the cancer presents both clinical and financial challenges 4, 12, 13. Facilities and resources in Canada to deal with end‐stage liver disease are generally inadequate aside from the few pockets of expertise at university‐based centers that have limited capacity 14. As HCC incidence increases, access to potentially curative treatments and the costs associated with them will present new challenges to the Canadian healthcare system. Important considerations include economic evaluation in general, and cost‐effectiveness analysis to help guide evidence‐based policy decision‐making in balancing health gains against the costs of interventions 15.

Net benefit regression framework utilizing person‐level data from administrative datasets facilitates the use of regression methods in the economic evaluation 16. Instead of the usual approach of aggregating cost and effect differences across different intervention strategies, the key advantage of the net benefit framework is the ability to use standard regression techniques, adjusting for explanatory variables to examine the marginal impact on incremental cost‐effectiveness 16. The net benefit calculation determines whether a new treatment meets (or surpasses) the decision‐maker's expectations of “good value for money”. The results can be used to help develop policy, with an aim toward improving efficiency and value in healthcare 17. We aimed to evaluate the real‐world cost‐effectiveness of potentially curative and combination (including TACE combined with curative treatment) treatments in patients diagnosed with HCC over a 9‐year study time frame from a healthcare payer's perspective in a Canadian setting.

## Materials and Methods

### Study design and population

The study included all eligible HCC cases aged 18 years and older in Ontario diagnosed between January 1, 2002 and December 31, 2010 to estimate the effectiveness, cost, and cost‐effectiveness of potentially curative treatments compared with no treatment to provide an estimate of the trade‐off between extra benefit and extra cost as well as utilizing net benefit regression framework to estimate the incremental net benefit (INB). HCC cases were identified through the Ontario Cancer Registry (OCR). The International Statistical Classification of Disease and Related Health Problems, 9th Revision (ICD‐9) site code 155.0, in combination with histology codes 8170–8175 of the International Classification of Diseases for Oncology, Third Edition (ICD‐O‐3), were used to identify cases of primary liver cancer. Patients who had death dates before or on the HCC diagnosis date during the study period were excluded. Furthermore, patients who received best supportive care within the first year after HCC diagnosis and those who received palliative treatments for advanced‐stage of HCC such as sorafenib or chemotherapy over the study period were also excluded.

### Data sources and study variables

The OCR is a provincial population‐based cancer registry that contains information on all new cases of cancer (except for nonmelanoma skin cancers) in Ontario since 1964 18. The OCR includes data regarding date and stage of HCC diagnosis, age, sex, birth location, urban or rural residence, cause of death, and date of death. The OCR cohort was linked to the Discharge Abstract Database maintained by the Canadian Institute for Health Information, the Ontario Health Insurance Plan (OHIP), the Ontario Drug Benefit Program, and the Canadian census data, to provide person‐level information on sociodemographic, screening, treatment, and clinical factors 19. The OHIP is a publically funded healthcare program for all Ontario residents; physician billing claims dataset contains service and diagnosis information for outpatient visits in Ontario. The Discharge Abstract Database contains information relating to diagnosis and procedures for all hospitalizations in Ontario, frequency and type of hospital admissions, length of stay, and in‐hospital mortality. The Ontario Drug Benefit dataset contains information regarding prescription medications (including sorafenib) dispensed to all adults aged 65 years and older and those receiving social assistance. Although there are some variances in different healthcare services, the system provides free access to hospital and emergency department visits, physician services, homecare, copayments for long‐term care placements, and prescription medications for those aged 65 years and older.

Area‐level socio‐economic status was quantified using median neighborhood household income. Median neighborhood household income was determined through linking of postal codes to Canadian census data; income was categorized into quintiles corresponding to income status of neighborhoods. The least and the most well‐off 20% of neighborhoods were included within the first and the fifth quintiles, respectively 20. Where possible, hospitalization records from the date of diagnosis were used to assign each patient and control subject a baseline Charlson–Deyo comorbidity index. If patients did not have a hospitalization record at their diagnosis date, baseline comorbidity was determined by looking back 2 years into the hospitalization data to find the most recent hospitalization record; the comorbidity score from that hospitalization was then applied 12, 13, 20, 21. The Charlson–Deyo comorbidity index at baseline was marked as “missing” if the individual had no hospitalization records at diagnosis or during the 2 years before diagnosis. Comorbidity was adjusted for each hospitalization after baseline. The Charlson–Deyo comorbidity index was calculated using methods previously described 22, 23; an ICD‐10 coding algorithm was applied to the diagnostic field codes from the hospitalization data (excluding diagnoses for liver disease, metastatic cancer, diabetes, and HIV). Conditions were weighted and then summed up to provide an overall comorbidity index value for a given episode, which was then categorized into one of five groups (0, 1, 2, ≥3, or no hospitalization record) representing different degrees of comorbidity.

Patients diagnosed with diabetes, HIV, and covariates that denote liver disease stage measured before HCC diagnosis were identified from the Discharge Abstract Database and OHIP using ICD‐9 and ICD‐10 codes. The study also included viral hepatitis cases identified through OHIP data; defined as subjects having at least two viral hepatitis visits (OHIP diagnostic code “070”) within the 4‐year interval before the HCC diagnosis date—to cover as much available OHIP data as possible. Indicators of liver disease stage were categorized exclusively as: (1) viral hepatitis; (2) no cirrhosis; (3) cirrhosis; (4) alcoholic liver disease (ALD) + cirrhosis; (5) viral hepatitis + cirrhosis; (6) ALD + viral hepatitis + cirrhosis; (7) decompensated cirrhosis (i.e., cirrhosis and any recorded ascites, esophageal varices, or hepatic encephalopathy); (8) ALD + decompensated cirrhosis; (9) nonalcoholic fatty liver disease (NAFLD) + decompensated cirrhosis; (10) viral hepatitis + decompensated cirrhosis; and (11) ALD + viral hepatitis + decompensated cirrhosis. Other relevant variables (including ALD alone, NAFLD alone, etc.) which were <20 in total were not considered as covariates.

To identify patients who received screening ultrasonography, we identified all abdominal ultrasonography performed on patients before HCC diagnosis utilizing OHIP fee codes 21. We obtained exclusive data regarding receipt of abdominal ultrasound screening (at least 4.5 months apart from previous ultrasound), which was defined as receiving one or more ultrasound screening annually for 2 years before HCC diagnosis (i.e., routine screening), at least one screen either within 12 months or between 12 and 24 months before HCC diagnosis (i.e., inconsistent screening), and no screening before HCC diagnosis.

### HCC treatment strategies

HCC treatments were identified from the Discharge Abstract Database, OHIP, and Ontario Drug Benefit databases determining the timing; for example, if both Discharge Abstract Database and OHIP for SR exits, we considered only the first SR. Mutually exclusive potentially curative monotherapies and combination therapies with palliative treatment (TACE) for HCC considered include: (i) RFA monotherapy; (ii) SR monotherapy; (iii) LT monotherapy; (iv) RFA plus SR; (v) RFA plus LT; (vi) SR plus LT; (vii) TACE plus RFA; (viii) TACE plus SR; (ix) TACE plus LT; (x) RFA plus SR plus LT triple treatment; and (xi) no treatment. Procedure codes used to identify diabetes, HIV, indicators of liver disease stage, HCC screening, and treatments can be found in the Tables S1 and S2 12, 20, 21.

### Measuring effectiveness

Life expectancy for each age in this study is the estimated average period that a person may expect to live, according to the age‐specific mortality rates for all causes. Potential years of life lost (PYLL, a measure of premature mortality) and quality‐adjusted life years lost (QALYL) were used to measure effectiveness. This study followed patients according to their death status until the end of year 2011. For those who died in or before 2011, age at death was calculated by adding years between diagnosis and death to the age at diagnosis. The age at diagnosis was recorded in the OCR cd‐link data as a categorical variable: below 60, 60–69, 70–79, or 80 years and above, which was assumed to be 55, 65, 75, or 85 years, respectively, in our analysis. To estimate age at death for patients who were still alive by the end of 2011, we first calculated the expected year of death based on the year of HCC diagnosis and the expected length of survival (i.e., period from diagnosis to death) according to stage at diagnosis; the estimate of survival was derived from the published literature (e.g., early‐stage I: 5 years; intermediate‐stage II: 4 years; and advanced‐stage III or IV: 3 years survival) 24, 25. If the expected year of death was 2011 or earlier, given the patient was still alive by the end of 2011, we assumed 2012 to be the most likely year of death. Accordingly, age at death could be estimated based on age at diagnosis and years between death and diagnosis. Subsequently, PYLL for each patient was determined using Ontario life tables which provided the standard life expectancy based on sex and age at death of an individual person 26.

QALYL consisted of two parts: (1) the PYLL was weighted by the average health state utility should the person be still alive and without disease; and (2) the number of years between diagnosis and death weighted by the quality of life according to the disease stage (from normal utility to utility of disease stage: noncirrhosis, compensated cirrhosis, decompensated cirrhosis, post‐LT or surgery in year 2 and onwards, and incurable HCC). The pooling of utility values for each stage (except incurable HCC = 0.40 [range: 0.32–0.48]) 27 was attempted using different preference‐based measures; these provide similar results by random effects models, and the estimates appeared to be close to other studies that provided input into decision‐analytic models 27, 28, 29. Although we developed the year‐specific model and considered treating stage as time‐dependent, only stage at diagnosis was available in the database; we could not obtain data regarding whether patients progressed beyond their disease stage at diagnosis. Pooled mean health state utilities of disease stage from published literature for base case analysis and the lower and upper bounds for sensitivity analyses are shown in Tables S3A–D.

### Measuring costs

Full details of data sources and estimation of direct health care costs associated with HCC has been previously published 12, 13. The total costs of healthcare services included outpatient visits, emergency department visits, acute inpatient hospitalizations, same‐day surgery, prescription medications, homecare visits, continuing care, and long‐term care. Costs associated with outpatient physician visits and laboratory tests in Ontario were estimated from the Physician Claims History Database of the OHIP. Costs for emergency department visits and same‐day surgery were estimated using the National Ambulatory Care Reporting System database 30. The costs of hospitalization, emergency department visits, and same‐day surgery for a particular year were estimated using the Resource Intensity Weight methodology developed by the Canadian Institute for Health Information 30. Prescription medication costs were obtained from the Ontario Drug Benefit Program 30. Costs associated with home care, continuing care, and long‐term care were estimated from the Ontario Home Care database, Continuing Care Reporting System, and Ontario Drug Benefit Program. Costs were adjusted for inflation to 2013 Canadian dollars using the Statistics Canada Consumer Price Index for healthcare and personal items for Ontario 31. Purchasing Power Parity for Gross Domestic Product was used to convert 2013 Canadian dollars to 2013 US dollars 32. Effects and costs were discounted at 3% annually as a base case to capture time preference given somewhat variation in the follow‐up time 33.

### Statistical analysis

Multiple imputation was used to impute values for variables with a high degree of missing data such as cancer stage at HCC diagnosis, birth country, and Charlson–Deyo comorbidity index. Five independent draws from an imputation model were used to create five completed datasets and results were combined to obtain one imputation inference 34. Statistically, multiple imputation is an established method to deal with replacing each missing value with a set of plausible values to ensure that the results are unbiased and capture the appropriate degree of precision 34. Multiple Imputation procedure by logistic regression was used in a sequential process to generate monotone patterns (PROC MI with LOGISTIC in the MONOTONE statement) 35, 36.

Next, we used the net benefit regression framework 16 to evaluate the real‐world cost‐effectiveness of curative treatments of HCC compared with no treatment among patients diagnosed with HCC from 2002 to 2010. In the first step, the net benefit value for each person (*NB*_*i*_) was calculated using the formula: willingness‐to‐pay (*λ*)*E_*i*_−*C*_*i*_, where *E*_*i*_ is the observed incremental effect (i.e., life year [LY] or quality‐adjusted life year [QALY] gained) and *C*_*i*_ is the incremental cost, for the *i*th person. Various values of *λ* for an additional effect 16 were explored ranging from $0 to $500,000. *NB*_*i*_ differs by various levels of *λ*; therefore, the person‐level net benefit is denoted as *NB(λ)*_*i*_.

The net benefit regression (i.e., multiple linear regression) involved fitting a linear regression model while adjusting for the relevant covariates (dummy variables), including sociodemographic characteristics: age (<60, 60–69, 70–79, ≥80 years), sex (male, female), income quintile (Q1‐lowest to Q5‐highest), residence (urban, rural), birth country (Canada, outside of Canada); clinical characteristics: Charlson–Deyo comorbidity index (0, 1, 2, ≥3), diabetes, HIV, liver disease stage, receipt of ultrasound screening 2 years before HCC diagnosis (routine screening, inconsistent screening, no screening), stage at diagnosis (early‐stage I, intermediate‐stage II, advanced‐stage III‐IV), and index year of HCC diagnosis (2002–2004, 2005–2007, 2008–2010). Additionally, we adjusted for propensity score to minimize bias related to the nonrandom allocation of potential curative treatment 37, 38. The propensity score for an individual is the conditional probability of assignment to having a curative treatment of HCC given the observed individual covariates. Here, it was derived by fitting a logistic regression model with HCC curative treatment as the dependent variable and the aforementioned covariates as independent variables. This approach allows for the adjustment of how covariates may affect the estimate of the intervention's INB (i.e., the marginal impact on incremental cost‐effectiveness, ICER) 16. The regression coefficient δ on the treatment dummy provides the estimate of the INB of treatment versus no treatment corresponding to a certain level of *λ* adjusted for the covariates. Treatment is defined to be cost‐effective, at a certain level of *λ*, if the corresponding INB is positive (i.e., INB >0).

Threshold values of the variance inflation factors were evaluated in the context of several other factors that influence the variance of regression coefficients 39. We eliminated interaction terms if there was no statistical significance or if the variance inflation factor values exceed 10 (i.e., indicating severe multicollinearity), which can reduce the variance of the regression coefficients. All covariates were included in the model because they were considered to be significant correlates of the outcome (theoretical justification) or were significantly different between the treatments (statistical validation).

The final step was assessing uncertainties and constructing cost‐effectiveness acceptability curves (CEACs) using the coefficient estimates of the treatment (*T*) variable and *P*‐values obtained from the net benefit regression model 16, 40. CEACs have been widely adopted as a method to quantify and incorporate the uncertainty that exists around the estimates of expected costs and expected effects associated with the interventions. A CEAC shows the probability that an intervention is cost‐effective compared with the alternative, given the observed data, for a range of *λ* values that a decision‐maker might be willing to pay for a particular unit change in the outcome (i.e., LY and QALY) 16, 40. The *P*‐values obtained from the net benefit regression are two‐sided but only one‐sided values are needed to test whether the INB is positive (i.e., treatment is cost‐effective) or negative (i.e., treatment is not cost‐effective) at the specified *λ*; therefore, the regression two‐sided *P*‐values are divided by two 16. For negative INB, the probability that the treatment is preferred equals the one‐sided *P*‐value, and for positive incremental net‐benefits, the probability that the treatment is preferred equals 1 minus the one‐sided *P*‐value 16. A CEAC was created by plotting a graphical representation that the HCC curative treatment is cost‐effective compared with no treatment (*y*‐axis), as a function of societal *λ* threshold per additional LY or QALY for a range of *λ* between $0 and $100,000 (*x*‐axis).

Analyses were performed using SAS version 9.4 (SAS Institute Inc., Cary, NC) and STATA version 12.0 (Stata Corporation, College Station, TX) statistical software applications.

### Ethics approval

Ethics approval for the study was granted by the University of Toronto Health Sciences Research Ethics Board. Informed consent was not obtained because this secondary analysis accessed existing de‐identified data; consent was therefore deemed to be neither feasible nor necessary.

## Results

### Description of cohort

Overall, 3,857 patients were identified as having a primary diagnosis of HCC from the OCR between 2002 and 2010. Flowchart of study population can be found in Figure S1. The final study cohort comprised 2,222 patients diagnosed with HCC after excluding 1,154 patients who had best supportive care within 1 year after HCC diagnosis, and 481 patients who had palliative treatments (chemotherapy and sorafenib) during the study period. The median and mean (standard deviation) of follow‐up time of patients diagnosed with HCC were 489 days and 735 (772) days, respectively. Overall baseline characteristics for this cohort are summarized in Table S4 and those stratified by treatment are summarized in Table 1. The majority (*n*= 1,182, 53.2%) of patients diagnosed with HCC did not receive curative treatment. Overall, 34.9% (*n*= 775) received a single curative treatment (10.5% received RFA, 14.1% SR and 10.3% LT monotherapy), 10.2% (*n*= 227) received dual treatments (3.1% received RFA plus SR, 2.5% RFA plus LT, 1.7% SR plus LT, 1.2% TACE plus RFA, 0.5% TACE plus SR, and 1.2% TACE plus LT), and 0.5% (*n*= 12) received triple treatments (RFA plus SR plus LT) during the study period.

**Table 1:** Baseline characteristics of patients with hepatocellular carcinoma by type of treatment, 2002–2010

Variable		Monotherapy			Dual treatments						Triple treatments
	No treatment	RFA	SR	LT	RFA + SR	RFA + LT	SR + LT	TACE + RFA	TACE + SR	TACE + LT	RFA + SR + LT
	n (%)	n (%)	n (%)	n (%)	n (%)	n (%)	n (%)	n (%)	n (%)	n (%)	n (%)
Overall	1182 (53.2)	234 (10.5)	313 (14.1)	228 (10.3)	69 (3.1)	55 (2.5)	37 (1.7)	27 (1.2)	12 (0.5)	27 (1.2)	12 (0.5)
Age group (years)											
<60	320 (27.1)	66 (28.2)	114 (36.4)	170 (74.6)	20 (29.0)	35 (63.6)	23 (62.2)	6 (22.2)	6 (50.0)	17 (63.0)	10 (83.3)
60–69	304 (25.7)	65 (27.8)	79 (25.2)	58 (25.4)	22 (31.9)	19 (34.6)	14 (37.8)	6 (22.2)	–	10 (37.0)	–
70–79	367 (31.1)	85 (36.3)	104 (33.2)	0	22 (31.9)	–	0	14 (51.9)	–	0	0
80 +	191 (16.2)	18 (7.7)	16 (5.1)	0	–	0	0	–	–	0	0
Sex											
Female	275 (23.3)	60 (25.6)	72 (23.0)	33 (14.5)	12 (17.4)	6 (10.9)	7 (18.9)	10 (37.0)	–	–	–
Male	907 (76.7)	174 (74.4)	241 (77.0)	195 (85.5)	57 (82.6)	49 (89.1)	30 (81.1)	17 (63.0)	9 (75.0)	26 (96.3)	10 (83.3)
Income quintile											
Q1 (lowest)	322 (27.2)	56 (23.9)	55 (17.6)	52 (22.8)	14 (20.3)	13 (23.6)	–	–	–	7 (25.9)	–
Q2	247 (20.9)	52 (22.2)	70 (22.4)	42 (18.4)	17 (24.6)	12 (21.8)	7 (18.9)	–	–	9 (33.3)	–
Q3	244 (20.6)	45 (19.2)	69 (22.0)	48 (21.1)	14 (20.3)	9 (16.4)	9 (24.3)	–	–	–	–
Q4	173 (14.6)	38 (16.2)	69 (22.0)	45 (19.7)	14 (20.3)	12 (21.8)	10 (27.0)	9 (33.3)	–	–	–
Q5 (highest)	185 (15.7)	42 (18.0)	50 (16.0)	41 (18.0)	9 (13.0)	9 (16.4)	6 (16.2)	–	0	6 (22.2)	0
Missing	11 (0.9)	–	0	0	–	0	0	0	0	0	0
Residence											
Urban	1061 (89.8)	220 (94.0)	288 (92.0)	207 (90.8)	67 (97.1)	52 (94.6)	33 (89.2)	27 (100)	12 (100)	26 (96.3)	12 (100)
Rural	118 (10.0)	14 (6.0)	25 (8.0)	21 (9.2)	–	–	–	0	0	–	0
Missing	–	0	0	0	0	0	0	0	0	0	0
Birth country											
Other	537 (45.4)	49 (20.9)	77 (24.6)	34 (14.9)	10 (14.5)	–	–	8 (29.6)	8 (66.7)	–	–
Canada	533 (45.1)	66 (28.2)	66 (21.1)	32 (14.0)	13 (18.8)	–	–	7 (25.9)	–	0	–
Unknown/missing	112 (9.5)	119 (50.9)	170 (54.3)	162 (71.1)	46 (66.7)	48 (87.3)	27 (73.0)	12 (44.4)	–	25 (92.6)	9 (75.0)
Comorbidity											
0	404 (34.2)	101 (43.2)	139 (44.4)	67 (29.4)	27 (39.1)	24 (43.6)	6 (16.2)	11 (40.7)	8 (66.7)	8 (29.6)	–
1	266 (22.5)	74 (31.6)	89 (28.4)	100 (43.9)	22 (31.9)	21 (38.2)	21 (56.8)	8 (29.6)	–	13 (48.2)	7 (58.3)
2	138 (11.7)	27 (11.5)	36 (11.5)	29 (12.7)	14 (20.3)	–	10 (27.0)	–	–	–	–
3 +	100 (8.5)	24 (10.3)	27 (8.6)	25 (11.0)	–	–	0	–	0	–	0
No hospitalization record	274 (23.2)	8 (3.4)	22 (7.0)	7 (3.1)	–	–	0	0	0	0	0
Diabetes	550 (46.5)	121 (51.7)	137 (43.8)	149 (65.4)	40 (58.0)	31 (56.4)	30 (81.1)	13 (48.2)	–	20 (74.1)	7 (58.3)
HIV	22 (1.9)	9 (3.9)	–	6 (2.6)	–	–	–	–	0	0	–
Indicators of liver disease stage											
Viral hepatitis	35 (3.0)	–	10 (3.2)	0	–	0	0	0	0	0	0
No cirrhosis	277 (23.4)	16 (6.8)	102 (32.6)	0	17 (24.6)	0	0	0	0	0	0
Cirrhosis	205 (17.3)	45 (19.2)	81 (25.9)	24 (10.5)	18 (26.1)	8 (14.6)	9 (24.3)	10 (37.0)	7 (58.3)	10 (37.0)	–
ALD + cirrhosis	41 (3.5)	13 (5.6)	6 (1.9)	–	–	–	0	0	0	0	0
Viral hepatitis + cirrhosis	38 (3.2)	10 (4.3)	18 (5.8)	–	–	–	–	0	0	0	–
ALD + Viral hepatitis + cirrhosis	6 (0.5)	–	–	0	–	–	0	–	0	0	0
Decompensated cirrhosis	288 (24.4)	70 (29.9)	61 (19.5)	85 (37.3)	12 (17.4)	18 (32.7)	14 (37.8)	11 (40.7)	–	12 (44.4)	6 (50.0)
ALD + decompensated cirrhosis	159 (13.5)	44 (18.8)	7 (2.2)	66 (29.0)	–	16 (29.1)	7 (18.9)	–	0	–	0
NAFLD + decompensated cirrhosis	8 (0.7)	–	0	–	–	0	–	0	0	–	0
Viral hepatitis + decompensated cirrhosis	53 (4.5)	14 (6.0)	12 (3.8)	19 (8.3)	–	–	–	–	–	0	0
ALD + viral hepatitis + decompensated cirrhosis	47 (4.0)	9 (3.9)	–	15 (6.6)	–	–	0	–	0	0	0
Ultrasound screening 2 years before HCC diagnosis											
No screening	622 (52.6)	86 (36.8)	125 (39.9)	113 (49.6)	24 (34.8)	24 (43.6)	9 (24.3)	9 (33.3)	–	6 (22.2)	–
Inconsistent screening	464 (39.3)	98 (41.9)	138 (44.1)	93 (40.8)	34 (49.3)	21 (38.2)	20 (54.1)	12 (44.4)	–	19 (70.4)	6 (50.0)
≥1 screens annually	96 (8.1)	50 (21.4)	50 (16.0)	22 (9.7)	11 (15.9)	10 (18.2)	8 (21.6)	6 (22.2)	–	–	–
Stage at HCC diagnosis											
Early (stage I)	66 (5.6)	62 (26.5)	65 (20.8)	35 (15.4)	18 (26.1)	12 (21.8)	6 (16.2)	8 (29.6)	–	6 (22.2)	–
Intermediate (stage II)	83 (7.0)	45 (19.2)	48 (15.3)	55 (24.1)	16 (23.2)	24 (43.6)	7 (18.9)	11 (40.7)	–	13 (48.2)	–
Advanced (stage III–IV)	274 (23.2)	10 (4.3)	51 (16.3)	12 (5.3)	6 (8.7)	–	–	–	–	–	0
Unknown	759 (64.2)	117 (50.0)	149 (47.6)	126 (55.3)	29 (42.0)	17 (30.9)	22 (59.5)	7 (25.9)	–	–	6 (50.0)
Year of HCC diagnosis											
2002	128 (10.8)	–	34 (10.9)	15 (6.6)	–	–	–	0	–	0	–
2003	106 (9.0)	10 (4.3)	33 (10.5)	23 (10.1)	7 (10.1)	0	–	–	–	0	–
2004	142 (12.0)	6 (2.6)	23 (7.4)	29 (12.7)	6 (8.7)	–	7 (18.9)	–	–	–	0
2005	135 (11.4)	14 (6.0)	47 (15.0)	28 (12.3)	–	–	–	–	–	–	–
2006	141 (11.9)	22 (9.4)	29 (9.3)	38 (16.7)	9 (13.0)	–	6 (16.2)	–	–	6 (22.2)	0
2007	142 (12.0)	19 (8.1)	34 (10.9)	32 (14.0)	7 (10.1)	6 (10.9)	–	–	0	–	–
2008	109 (9.2)	46 (19.7)	25 (8.0)	23 (10.1)	13 (18.8)	18 (32.7)	–	6 (22.2)	0	–	0
2009	137 (11.6)	56 (23.9)	45 (14.4)	21 (9.2)	10 (14.5)	10 (18.2)	0	–	–	–	–
2010	142 (12.0)	59 (25.2)	43 (13.7)	19 (8.3)	9 (13.0)	12 (21.8)	–	7 (25.9)	0	–	0

“–”, counts less than six have been suppressed.

RFA, radiofrequency ablation; SR, surgical resection; LT, liver transplantation; HCC, hepatocellular carcinoma.

With regard to dual treatments with RFA, 46 (67%) patients underwent primary SR; with LT, 52 (95%) underwent primary RFA, 12 (32%) underwent primary SR, and 27 (100%) underwent primary TACE; with TACE, 17 (63%) underwent primary RFA and 12 (100%) underwent primary SR. Of the 2,222 patients, 13.0% were stage I, 14.2% stage II, 13.0% stage III, 3.7% stage IV, and 56.2% unknown stage at diagnosis. Patients with unknown stage were less likely to have received curative treatments. Age (except RFA + SR and TACE + SR), birth country (except SR and TACE + RFA), Charlson–Deyo comorbidity index (except TACE + RFA, TACE + SR, TACE + LT, and RFA + SR + LT), liver disease stage (except SR + LT, TACE + RFA, TACE + LT, and RFA + SR + LT), and cancer stage (except SR + LT, TACE + SR, and RFA + SR + LT) were associated with receipt of curative treatments (*P *< 0.05); additionally, sex was associated with receipt of LT, RFA + LT or TACE + LT (*P *< 0.05), diabetes was associated with receipt of SR, LT, SR + LT or TACE + LT (*P *< 0.05), ultrasound screening was associated with receipt of RFA, SR, SR + LT or TACE + LT (*P *< 0.05), and year of HCC diagnosis was associated with receipt of RFA, SR, LT, RFA + LT or RFA + SR + LT (*P *< 0.05).

### Healthcare effects and costs

Effects and costs stratified by treatment strategies are summarized in Table 2. The lowest QALYL was among those who received RFA + SR (9.1, 95% confidence interval [CI]: 8.8–9.5) and the highest QALYL were among those who received LT (11.9, 95% CI: 11.7–12.0) or RFA + LT (11.8, 95% CI: 11.6–12.1). The lowest costs were among those who did not receive treatment ($38,472, 95% CI: $37,255–$39,689) followed by those who received TACE + RFA ($48,485, 95% CI: $43,663–$53,307), and RFA monotherapy ($55,925, 95% CI: $52,123–$59,727); and the highest costs were among those who received SR + LT ($222,275, 95% CI: $205,992–$238,558) or RFA + SR + LT ($208,484, 95% CI: $190,385–$226,582).

**Table 2:** Healthcare effects and costs after diagnosis of hepatocellular carcinoma by treatment strategies, 2002–2010

Treatment strategies	Effects (mean, 95% CI)		Costs^a^ (mean, 95% CI)
	PYLL	QALYL	
No treatment (*n*= 5910)	11.2251 (11.1105–11.3397)	10.1149 (10.009–10.2207)	$38,472 ($37,255–$39,689)
Monotherapy			
RFA (*n*= 1170)	10.2246 (9.9959–10.4534)	9.8759 (9.6697–10.0821)	$55,925 ($52,123–$59,727)
SR (*n*= 1565)	10.0818 (9.8927–10.2709)	9.9144 (9.7466–10.0822)	$119,032 ($115,799–$122,265)
LT (*n*= 1140)	11.9376 (11.7841–12.0911)	11.8696 (11.7471–11.992)	$211,286 ($203,566–$219,007)
Dual treatments			
TACE plus RFA (*n*= 135)	9.6379 (8.979–10.2968)	9.3606 (8.7722–9.9489)	$48,485 ($43,663–$53,307)
RFA plus SR (*n*= 345)	9.0966 (8.6997–9.4934)	9.1399 (8.7844–9.4953)	$109,927 ($103,953–$115,902)
TACE plus SR (*n*= 60)	11.4624 (10.4676–12.4573)	10.999 (10.0909–11.907)	$126,514 ($114,451–$138,577)
RFA plus LT (*n*= 275)	12.0635 (11.7867–12.3402)	11.8248 (11.5797–12.0699)	$155,898 ($144,119–$167,677)
TACE plus LT (*n*= 135)	11.4675 (11.0773–11.8578)	11.4621 (11.1359–11.7883)	$178,354 ($163,494–$193,215)
SR plus LT (*n*= 185)	10.756 (10.3731–11.1388)	10.8734 (10.5621–11.1847)	$222,275 ($205,992–$238,558)
Triple treatments			
RFA plus SR plus LT (*n*= 60)	11.1088 (10.3933–11.8242)	11.2472 (10.6504–11.844)	$208,484 ($190,385–$226,582)

RFA, radiofrequency ablation; SR, surgical resection; LT, liver transplantation; PYLL, potential years of life lost (a measure of premature mortality); QALYL, quality‐adjusted life years lost.

a All costs reflect 2013 US$ per person. Multiple imputation by logistic regression was used to generate missing data (cancer stage at HCC diagnosis, birth country, and Charlson–Deyo comorbidity index) for outcomes.

### Net benefit regression

**Figure 1:**
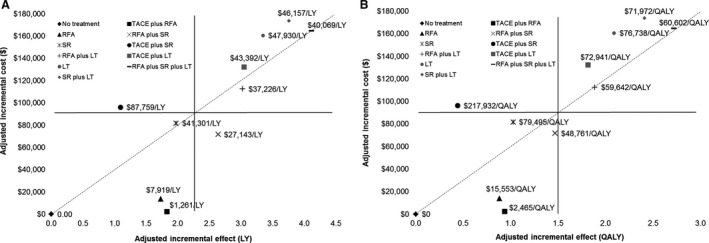
Efficiency frontier: plot of incremental (A) life years (LYs) and (B) quality‐adjusted life years (QALYs) and costs of curative treatments relative to lowest cost scenario (no treatment). The dotted diagonal line represents the willingness‐to‐pay for health effects (maximum acceptable ceiling ratio). If an intervention lies above the line, it will not be acceptable on cost‐effectiveness grounds.

Compared with no treatment (adjusted for important covariates), LT‐related treatments were estimated to yield more units of QALYs (incremental QALYs: RFA + SR + LT=2.72; SR + LT=2.41; LT monotherapy=2.09; RFA + LT=1.88; and TACE + LT=1.81); but more costly ($164,608, $173,575, $160,430, $112,411, and $132,266, respectively) than RFA + SR, SR monotherapy, TACE + RFA, RFA monotherapy or TACE + SR (incremental QALYs: 1.47, 1.03, 0.93, 0.88, and 0.44, respectively; incremental costs: $71,559, $81,514, $2,304, $13,697, and $96,088, respectively)(Table 3). In Figure 1A and B plot of incremental LYs and QALYs and costs of curative treatments relative to lowest cost scenario (no treatment), TACE plus RFA, RFA monotherapy, and RFA + SR dual treatment below the line (dotted diagonal line representing the ceiling ratio) appeared to be acceptable.

**Table 3:** Effects, costs, and incremental cost‐effectiveness ratios of potentially curative treatment strategies for hepatocellular carcinoma compared with no treatment, 2002–2010: net benefit regression

Treatment strategies	Average total effect (PYLL)	Average total effect (QALYL)	Average total cost ($)	Adj incr effect^a^ (LYs)	Adj incr effect^`^ a (QALYs)	Adj incr cost ($)^b^	Adj ICER ($/LY gained)	Adj ICER ($/QALY gained)
No treatment	11.2251	10.1149	$38,472					
TACE plus RFA	9.6379	9.3606	$48,485	1.82717	0.93455	$2,304	$1,261	$2,465
RFA	10.2246	9.8759	$55,925	1.72958	0.88067	$13,697	$7,919	$15,553
RFA plus SR	9.0966	9.1399	$109,927	2.63641	1.46756	$71,559	$27,143	$48,761
SR	10.0818	9.9144	$119,032	1.97368	1.02540	$81,514	$41,301	$79,495
TACE plus SR	11.4624	10.999	$126,514	1.09491	0.44091	$96,088	$87,759	$217,932
RFA plus LT	12.0635	11.8248	$155,898	3.01967	1.88475	$112,411	$37,226	$59,642
TACE plus LT	11.4675	11.4621	$178,354	3.04816	1.81332	$132,266	$43,392	$72,941
RFA plus SR plus LT	11.1088	11.2472	$208,484	4.10809	2.71620	$164,608	$40,069	$60,602
LT	11.9376	11.8696	$211,286	3.34719	2.09062	$160,430	$47,930	$76,738
SR plus LT	10.756	10.8734	$222,275	3.76051	2.41171	$173,575	$46,157	$71,972

Values are expressed as the mean. All costs reflect 2013 US$ per person.

RFA, radiofrequency ablation; SR, surgical resection; LT, liver transplantation; PYLL, potential years of life lost; QALYL, quality‐adjusted life years lost; LY, life year.

a Incremental effect is calculated as treatment effect minus no treatment effect, adjusted for relevant covariates (dummy variables), including age, sex, income quintile, urban/rural residence, birth country, Charlson–Deyo comorbidity index, diabetes, HIV, indicators of liver disease stage, ultrasound screening, stage at HCC diagnosis, and year of HCC diagnosis. Positive value indicates increase in the effect relative to “no treatment”.

b Incremental cost is calculated as treatment cost minus no treatment cost, adjusted for aforementioned covariates. Positive value indicates increase in cost relative to “no treatment”.

**Figure 2:**
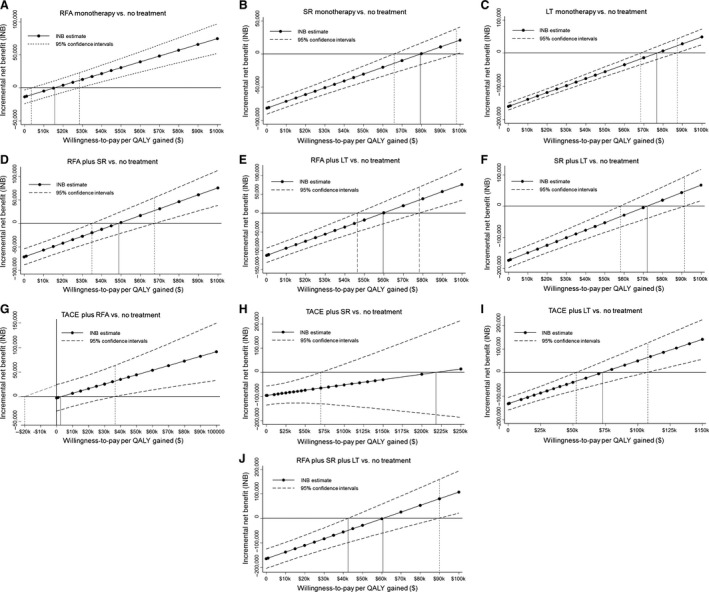
Estimates of incremental net benefit (i.e., incremental cost‐effectiveness ratio, ICER) and its 95% confidence intervals as a function of willingness‐to‐pay threshold for an additional quality‐adjusted life year (QALY); (A) radiofrequency ablation (RFA) monotherapy versus no treatment; (B) surgical resection (SR) monotherapy versus no treatment; (C) liver transplantation (LT) monotherapy versus no treatment; (D) RFA plus SR versus no treatment; (E) RFA plus LT versus no treatment; (F) SR plus LT versus no treatment; (G) TACE plus RFA versus no treatment; (H) TACE plus SR versus no treatment; (I) TACE plus LT versus no treatment; and (J) RFA plus SR plus LT versus no treatment.

Figure S2A–J (LYs) and Figure 2A–J (QALYs) show estimates of INB (i.e., ICER estimate) and its 95% CIs as a function of willingness‐to‐pay thresholds. The lowest ICER estimate for TACE plus RFA was $2,465/QALY gained (95% CI: −$20,000‐$36,600/QALY); this means TACE plus RFA is cost‐effective if decision‐makers value >$36,600/QALY threshold, but not cost‐effective if decision‐makers value <−$20,000/QALY threshold. Alternative ICER estimates in order were: for RFA monotherapy, $15,553/QALY (95% CI: $3,500–$28,500/QALY); RFA + SR, $48,761/QALY (95% CI: $35,000–$67,200/QALY); RFA + LT, $59,642/QALY (95% CI: $46,600–$78,000/QALY); RFA + SR + LT, $60,602/QALY (95% CI: $42,600–$90,000/QALY); SR + LT, $71,972/QALY (95% CI: $58,100–$91,450/QALY); TACE + LT, $72,941/QALY (95% CI: $52,700–$107,600/QALY); LT monotherapy, $76,738/QALY (95% CI: $68,400–$87,200/QALY); SR only, $79,495/QALY (95% CI: $65,800–$98,200/QALY); and TACE + SR, $217,932/QALY (95% CI: LB $70,000/QALY; UB undefined) .

**Figure 3:**
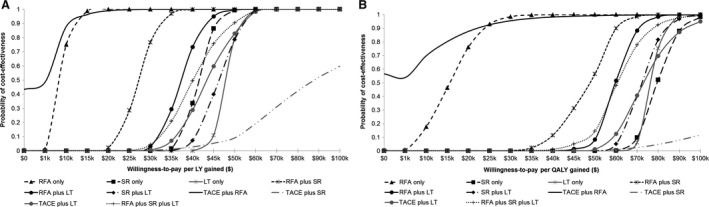
Cost‐effectiveness acceptability curves showing the probability that each curative treatment strategy is cost‐effective compared with no treatment for a given willingness‐to‐pay threshold for an additional (A) life year (LY); and (B) quality‐adjusted life year (QALY).

Figure 3A and B show CEACs which plot the probability that each treatment strategy is cost‐effective compared with no treatment as a function of willingness‐to‐pay threshold for an additional LY and QALY, respectively. The results showed that if a threshold of $50,000/LY gained was to be chosen, RFA monotherapy, SR monotherapy, RFA plus SR, RFA plus LT, and TACE plus RFA would have a cost‐effectiveness probability of 99–100% (Table S5); whereas, if $50,000/QALY gained was to be chosen, RFA monotherapy and TACE plus RFA would have a cost‐effectiveness probability of 99–100% (Table 4); and if a threshold of $100,000/QALY gained was to be chosen, all treatments would have a cost‐effectiveness probability of more than 95% (except TACE plus SR [12%], Table 4).

**Table 4:** Estimates of incremental net benefit and probability of cost‐effectiveness of curative treatment strategies for hepatocellular carcinoma compared with no treatment as a function of willingness‐to‐pay threshold per additional QALY over the study period 2002–2010

*λ* thresholds	Radiofrequency ablation			Surgical resection			Liver transplantation		
	INB estimate (SE)	*P*‐value^a^	Probability of cost‐effectiveness	INB estimate (SE)	*P*‐value^a^	Probability of cost‐effectiveness	INB estimate (SE)	*P*‐value^a^	Probability of cost‐effectiveness
$0	−13,698 (5332)	0.005	0.0051	−81,536 (4592)	<0.001	0.0001	−160,428 (5568)	<0.001	0.0001
$1,000	−12,817 (5319)	0.008	0.008	−80,511 (4582)	<0.001	0.0001	−158,337 (5554)	<0.001	0.0001
$10,000	−4892 (5300)	0.178	0.1781	−71,287 (4580)	<0.001	0.0001	−139,516 (5534)	<0.001	0.0001
$20,000	3915 (5487)	0.238	0.7622	−61,037 (4755)	<0.001	0.0001	−118,605 (5731)	<0.001	0.0001
$30,000	12,721 (5872)	0.015	0.9849	−50,787 (5099)	<0.001	0.0001	−97,693 (6138)	<0.001	0.0001
$40,000	21,527 (6420)	<0.001	0.9996	−40,537 (5580)	<0.001	0.0001	−76,781 (6717)	<0.001	0.0001
$50,000	30,333 (7093)	<0.001	1.0000	−30,288 (6167)	<0.001	0.0001	−55,870 (7427)	<0.001	0.0001
$60,000	39,139 (7859)	<0.001	1.0000	−20,038 (6832)	0.002	0.0017	−34,958 (8235)	<0.001	0.0001
$70,000	47,945 (8693)	<0.001	1.0000	−9788 (7555)	0.098	0.0976	−14,047 (9114)	0.062	0.0617
$80,000	56,751 (9578)	<0.001	1.0000	462 (8320)	0.478	0.5221	6865 (10,047)	0.247	0.7528
$90,000	65,557 (10,501)	<0.001	1.0000	10,711 (9117)	0.120	0.8800	27,777 (11,019)	0.006	0.9942
$100,000	74,363 (11,452)	<0.001	1.0000	20,961 (9938)	0.017	0.9826	48,688 (12,020)	<0.001	1.0000

*λ* thresholds	Radiofrequency ablation plus surgical resection			Radiofrequency ablation plus liver transplantation			Surgical resection plus liver transplantation		
	INB estimate (SE)	*P*‐value^a^	Probability of cost‐effectiveness	INB estimate (SE)	*P*‐value^a^	Probability of cost‐effectiveness	INB estimate (SE)	*P*‐value^a^	Probability of cost‐effectiveness
$0	−71,514 (8749)	<0.001	0.0001	−112,421 (9864)	<0.001	0.0001	−173,510 (11,812)	<0.001	0.0001
$1,000	−70,046 (8730)	<0.001	0.0001	−11,0538 (9842)	<0.001	0.0001	−171,100 (11,789)	<0.001	0.0001
$10,000	−56,830 (8726)	<0.001	0.0001	−93,589 (9836)	<0.001	0.0001	−149,406 (11,800)	<0.001	0.0001
$20,000	−42,146 (9063)	<0.001	0.0001	−74,757 (10,214)	<0.001	0.0001	−125,302 (12,268)	<0.001	0.0001
$30,000	−27,462 (9721)	0.002	0.0024	−55,926 (10,957)	<0.001	0.0001	−101,197 (13,166)	<0.001	0.0001
$40,000	−12,778 (10,642)	0.115	0.1149	−37,094 (11,998)	0.001	0.0010	−77,093 (14,414)	<0.001	0.0001
$50,000	1905 (11,764)	0.436	0.5644	−18,262 (13,266)	0.0843	0.0843	−52,989 (15,929)	<0.001	0.0005
$60,000	16,589 (13,035)	0.102	0.8985	570 (14,703)	0.48455	0.5155	−28,885 (17,644)	0.051	0.0508
$70,000	31,273 (14,415)	0.015	0.9850	19,402 (16,264)	0.11645	0.8836	−4781 (19,505)	0.403	0.4032
$80,000	45,957 (15,877)	0.002	0.9981	38,233 (17,916)	0.0164	0.9836	19,324 (21,474)	0.184	0.8159
$90,000	60,641 (17,399)	<0.001	0.9998	57,065 (19,638)	0.00185	0.9982	43,428 (23,524)	0.032	0.9676
$100,000	75,325 (18,968)	<0.001	1.0000	75,897 (21,411)	0.0002	0.9998	67,532 (25,637)	0.004	0.9958

*λ* thresholds	TACE plus radiofrequency ablation			TACE plus surgical resection			TACE plus liver transplantation		
	INB estimate (SE)	*P*‐value^a^	Probability of cost‐effectiveness	INB estimate (SE)	*P*‐value^a^	Probability of cost‐effectiveness	INB estimate (SE)	*P*‐value^a^	Probability of cost‐effectiveness
$0	−2262 (13,639)	0.434	0.5659	−95,930 (20,116)	<0.001	0.0001	−132,378 (13,697)	<0.001	0.0001
$1,000	−1326 (13,611)	0.461	0.5388	−95,491 (20,076)	<0.001	0.0001	−130,562 (13,668)	<0.001	0.0001
$10,000	7094 (13,612)	0.301	0.6989	−91,538 (20,096)	<0.001	0.0001	−114,221 (13,670)	<0.001	0.0001
$20,000	16,449 (14,146)	0.122	0.8776	−87,145 (20,893)	<0.001	0.0001	−96,065 (14,207)	<0.001	0.0001
$30,000	25,804 (15,183)	0.045	0.9554	−82,753 (22,420)	<0.001	0.0001	−77,909 (15,248)	<0.001	0.0001
$40,000	35,160 (16,628)	0.017	0.9828	−78,360 (24,541)	<0.001	0.0007	−59,752 (16,699)	<0.001	0.0002
$50,000	44,515 (18,386)	0.008	0.9923	−73,968 (27,118)	0.003	0.0032	−41,596 (18,463)	0.012	0.0122
$60,000	53,870 (20,376)	0.004	0.9959	−69,575 (30,032)	0.010	0.0103	−23,439 (20,461)	0.126	0.1260
$70,000	63,226 (22,536)	0.003	0.9975	−65,182 (33,196)	0.025	0.0248	−5283 (22,629)	0.408	0.4077
$80,000	72,581 (24,822)	0.002	0.9983	−60,790 (36,544)	0.048	0.0481	12,874 (24,924)	0.303	0.6973
$90,000	81,936 (27,203)	0.001	0.9987	−56,397 (40,030)	0.079	0.0795	31,030 (27,314)	0.128	0.8721
$100,000	91,292 (29,655)	0.001	0.9990	−52,005 (43,622)	0.117	0.1166	49,187 (29,775)	0.049	0.9508

*λ* thresholds	Radiofrequency ablation plus surgical resection plus liver transplantation		
	INB estimate (SE)	*P*‐value^a^	Probability of cost‐effectiveness
$0	−164,675 (20,178)	<0.001	0.0001
$1,000	−161,956 (20,138)	<0.001	0.0001
$10,000	−137,487 (20,158)	<0.001	0.0001
$20,000	−110,300 (20,961)	<0.001	0.0001
$30,000	−83,113 (22,500)	<0.001	0.0001
$40,000	−55,925 (24,636)	0.012	0.0116
$50,000	−28,738 (27,230)	0.146	0.1457
$60,000	−1551 (30,164)	0.480	0.4795
$70,000	25,637 (33,348)	0.221	0.779
$80,000	52,824 (36,717)	0.075	0.925
$90,000	80,011 (40,225)	0.023	0.977
$100,000	107,199 (43,838)	0.007	0.993

*λ*, willingness‐to‐pay; INB, incremental net benefit; SE, standard error.

a One‐sided *P*‐value.

### Sensitivity analysis

Sensitivity analysis according to the pooled lower and upper bound health state utilities of disease stage from published literature showed that TACE plus RFA, RFA monotherapy, and RFA + SR dual treatment appeared to be acceptable compared to no treatment, similar to base case (Fig. S3A and B). A sensitivity analysis of excluding HCC stage IV appeared robust to the base case (stage IV was lumped with stage III) relating to the incremental effects (LYs and QALYs) and costs, and ICER of curative treatments relative to no treatment (Fig. S4A and B).

## Discussion

This study evaluated the real‐world cost‐effectiveness of potentially curative treatments compared with no treatment among patients diagnosed with HCC over a 9‐year study time frame from a healthcare payer's perspective in Ontario, Canada's most populated province with approximately 13.6 million people as of year 2013. We note that during this time period, no new curative therapies have become available. Compared with no treatment, the adjusted incremental benefit of LT‐related treatments are estimated to yield more units of QALYs than RFA or SR treatments, but are more costly. ICERs of TACE plus RFA dual treatment (i.e., major primary RFA; average $2,465, 95% CI: −$20,000–$36,600/QALY gained) and RFA monotherapy (average $15,553, 95% CI: $3,500–$28,500/QALY gained) are below the commonly cited thresholds of $50,000/QALY 41. Interventions costing less than $50,000/QALY are often considered cost‐effective 42, but oncologists commonly endorse higher thresholds 43. The CEACs show that if a threshold of $50,000/QALY gained is to be chosen, RFA monotherapy and TACE plus RFA would have a cost‐effectiveness probability of 99–100%. If a threshold of $100,000/QALY gained is to be chosen, all treatments would have a cost‐effectiveness probability of more than 95% (except TACE plus SR).

Historically, percutaneous RFA was widely used for local control of small unresectable HCC including those patients who could not tolerate SR but it is increasingly being used as first‐line therapy for patients with amenable lesions 9. Based on published papers, the combination treatment of TACE and RFA seems to be a safe and effective treatment strategy for patients with early‐ or intermediate‐stage HCC 44, 45. In patients included in the waiting list for a LT, tumors are often treated as a “bridge” to transplant while waiting for an organ to become available because of the risk of tumor progression. RFA is widely used as a “bridge” to transplantation in order to avoid this progression 46. This analysis was able to include patients who may have undergone palliative treatment (i.e., TACE) as a bridge to LT.

A few other studies analyzed the cost‐effectiveness of two potentially curative treatment strategies in early‐stage HCC within the Milan criteria using Markov cohort models. Cucchetti et al. compared SR with RFA and found that in the presence of two or three nodules ≤3 cm, RFA is more cost‐effective than resection; for single larger early‐stage HCC, SR is cost‐effective at a willingness‐to‐pay largely acceptable in the oncologic setting 27. Lim et al. compared SR with LT and found that in patients with HCC within the Milan criteria and Child‐Pugh A/B cirrhosis, SR is more cost‐effective than cadaveric LT across three different costing scenarios: the USA, Switzerland, and Singapore 28. Our findings highlight the important implications for identification and measurement of differential benefits, costs, and cost‐effectiveness of alternative HCC curative treatments in order to evaluate whether particular healthcare technologies are providing good value for money in the real‐world within the context of an organized healthcare system.

HCC is a rapidly rising disease and many patients who develop HCC present late which becomes an incurable advanced‐stage disease. The biggest limitation to treating HCC is the function of the underlying liver and the stage of first HCC diagnosis. It is evident that patients with HCC receiving treatments might appear fairly different with regard to clinical and tumor features that are known to affect prognosis 47. Patients might not be considered suitable for surgery because of liver dysfunction and/or portal hypertension, as well as the presence of comorbidities or advanced age contraindicating general anesthesia 47. Screening and surveillance programs for early detection of HCC and keeping track of the outcomes for quality assurance are needed 48. Given the increasing and high mortality rate associated with HCC, investments and healthcare resources at existing regional cancer centers should be enhanced to facilitate the multidisciplinary care to mitigate the impact of the disease 14, 49. Thus, treating liver disease is a priority and is needed to act to protect the health and well‐being of Canadians of all ages. Recommendations to improve the control of HCC in Canada include healthcare providers to identify, offer testing, and counsel people at risk for HCC such as alcohol‐and non‐alcohol‐induced liver disease, cirrhosis, diabetes, obesity, smoking, 50, 51, 52, 53, 54 and HIV coinfection 55, in particular, marginalized groups of people. Furthermore, patients need easier access to treatments to reduce the chance of progression to liver cancer.

The net benefit framework can clearly demonstrate how different the ICERs are when adjusted or not adjusted for covariates. The advantage of this is that influential covariates can be adjusted for in the regression model to obtain a more accurate INB 16. This NBR found several covariates associated with INB (*P *< 0.05), including age group and sex (from *λ* $10,000 to *λ* $100,000), income quintile (from *λ* $0 to *λ* $25,000), Charlson–Deyo comorbidity index, cirrhosis, and viral hepatitis + cirrhosis (from *λ* $0 to *λ* $100,000), ALD + cirrhosis, decompensated cirrhosis, and routine screening (i.e., one or more ultrasound screening annually for 2 years before HCC diagnosis) (from *λ* $0 to *λ* $50,000), and HIV diagnosis and year of HCC diagnosis (from *λ* $25,000 to *λ* $100,000). The strategy with the largest incremental benefit while still being cost‐effective may be the preferred strategy.

This study is meant to be a cost‐effectiveness analysis taken from the perspective of the healthcare payer on a population‐level (and not an individual‐level decision‐analysis to aid individual decision‐making). This helps payers understand the trade‐offs of cost and benefits of the various curative intent therapies. This information can help with priority‐setting to allow payers maximize health outcomes if they are faced with finite budget. Given the rising cost of therapies, especially with the rising cost of new expensive drugs, this study can help payers understand the value for money of the curative intent therapies in the context of demands to fund newer but palliative expensive drugs.

There are a number of limitations in this study that should be considered. Using a 9‐year observation period may result in an underestimation of the true benefits of HCC curative treatment. If HCC curative treatment does have an impact on the long‐term survival, the full impact will not be observed within the 9‐year observation period. Although additional work such as building a mathematical model is not within the scope of this study, data collected can be utilized to support future modeling studies. Other limitations include the stage of HCC at treatment; clearly, smaller lesions detected early by regular serial ultrasound surveillance, will be associated with lower costs and better survival, than more advanced lesions requiring multi‐modal treatment or LT. From our analysis, it can be inferred that ultrasound surveillance programs may be associated with lower costs and better cost‐effectiveness if small lesions amenable to RFA only, can be diagnosed. This may have an effect but overall, there is only 11.8% receiving regular ultrasound screening. Patients who may have undergone RFA at laparotomy (i.e., because of location of the HCC) may have been coded as dual therapy or SR clinically.

## Conclusions

From our analysis, compared with no treatment, TACE plus RFA dual treatment or RFA monotherapy appears to be the most cost‐effective modality with lowest ICER value if a threshold of $50,000/QALY gained was to be chosen, but this is generalizable only to those who are diagnosed with early HCC lesions. In order to achieve optimal cost‐effectiveness of HCC treatment with curative intent, patients at risk of HCC must be diagnosed early (i.e., via regular ultrasound surveillance) and referred for treatment in a timely manner before HCC disease progression requires more advanced curative treatment (i.e., SR, LT, etc.) that is associated with greater healthcare costs and less favorable cost‐effectiveness calculations.

## Conflict of Interest

The authors declare that they have no competing interests.

## Supporting information


**Figure S1.** Selection criteria for the study sample.

**Figure S2.** Estimates of incremental net benefit (i.e., incremental cost‐effectiveness ratio, ICER) and its 95% confidence intervals as a function of willingness‐to‐pay threshold for an additional life year (LY); (S2A) radiofrequency ablation (RFA) monotherapy versus no treatment; (S2B) surgical resection (SR) monotherapy versus no treatment; (S2C) liver transplantation (LT) monotherapy versus no treatment; (S2D) RFA plus SR versus no treatment; (S2E) RFA plus LT versus no treatment; (S2F) SR plus LT versus no treatment; (S2G) TACE plus RFA versus no treatment; (S2H) TACE plus SR versus no treatment; (S2I) TACE plus LT versus no treatment; and (S2J) RFA plus SR plus LT versus no treatment.

**Figure S3.** Efficiency frontier: plot of incremental quality‐adjusted life years (QALYs) and costs of curative treatments relative to lowest cost scenario (no treatment): Sensitivity analysis according to (S3A) lower bound and (S3B) upper bound of pooled mean health state utilities of disease stage from published literature. The dotted diagonal line represents the willingness‐to‐pay for health effects (maximum acceptable ceiling ratio). If an intervention lies above the line, it will not be acceptable on cost‐effectiveness grounds.

**Figure S4.** Efficiency frontier: Efficiency frontier: plot of incremental (A) life years (LYs) and (B) quality‐adjusted life years (QALYs) and costs of curative treatments relative to lowest cost scenario (no treatment): Sensitivity analysis of excluding HCC stage IV.

**Tables S1.** ICD‐9 and ICD‐10 codes for patients diagnosed with diabetes, HIV, and liver disease stage.
**Table S2.** Screening and treatment procedures for patients with hepatocellular carcinoma.
**Table S3.** Estimation of utilities for noncirrhosis.
**Table S4.** Baseline characteristics of patients with hepatocellular carcinoma, 2002–2010.
**Table S5.** Estimates of incremental net benefit and probability of cost‐effectiveness of curative treatment strategies for hepatocellular carcinoma compared with no treatment as a function of willingness‐to‐pay threshold per additional life year over the study period 2002–2010.
