# Supplementary figures and images for: Cost‐effectiveness analysis of potentially curative and combination treatments for hepatocellular carcinoma with person‐level data in a Canadian setting
Source: Cancer Med. 2017 Aug 8;6(9):2017–33. doi: 10.1002/cam4.1119 (PMC5603843; doi:10.1002/cam4.1119)

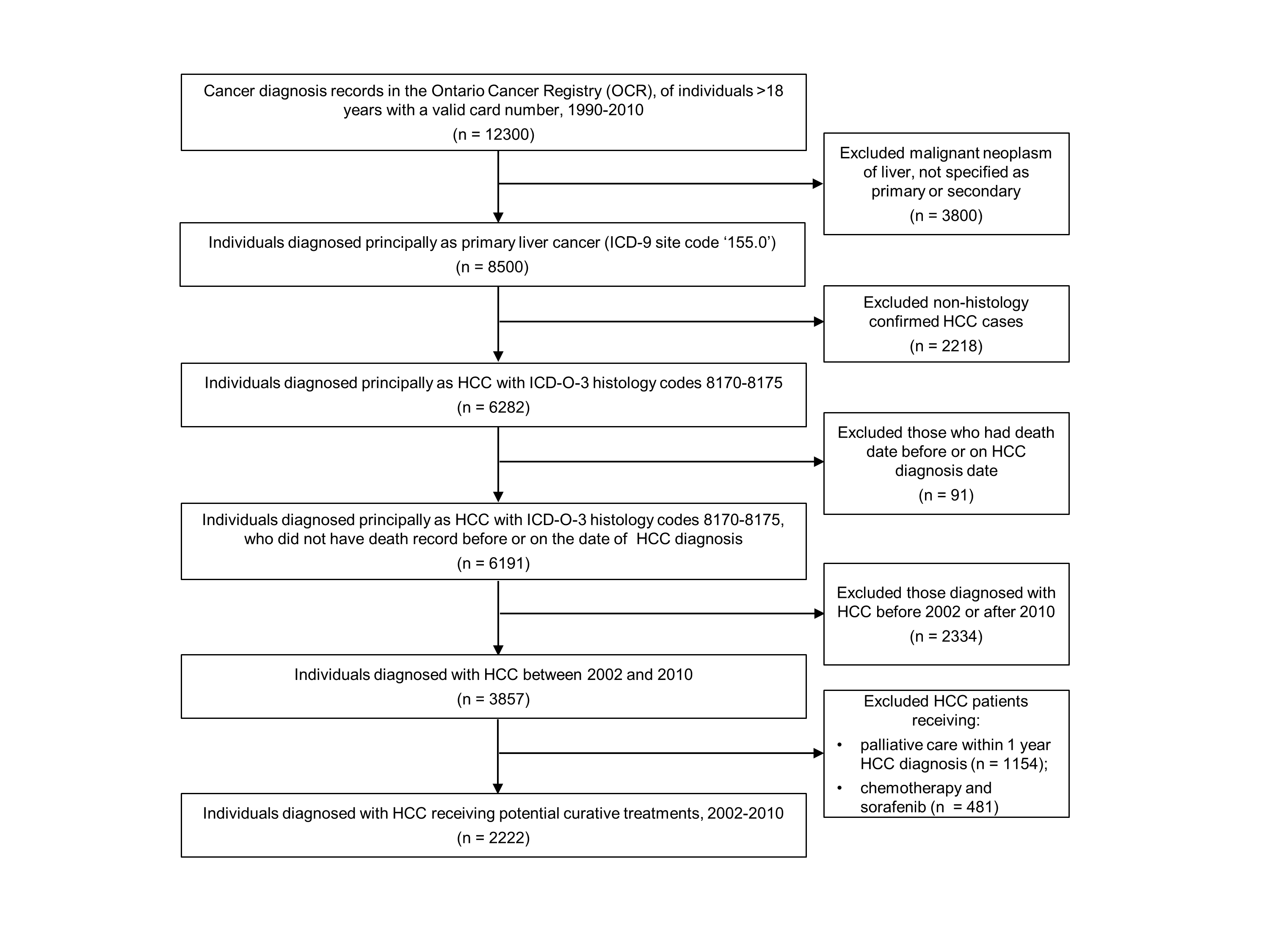

Supplement: Supplementary file 1 — Figure S1. Selection criteria for the study sample. [file CAM4-6-2017-s001.tif]

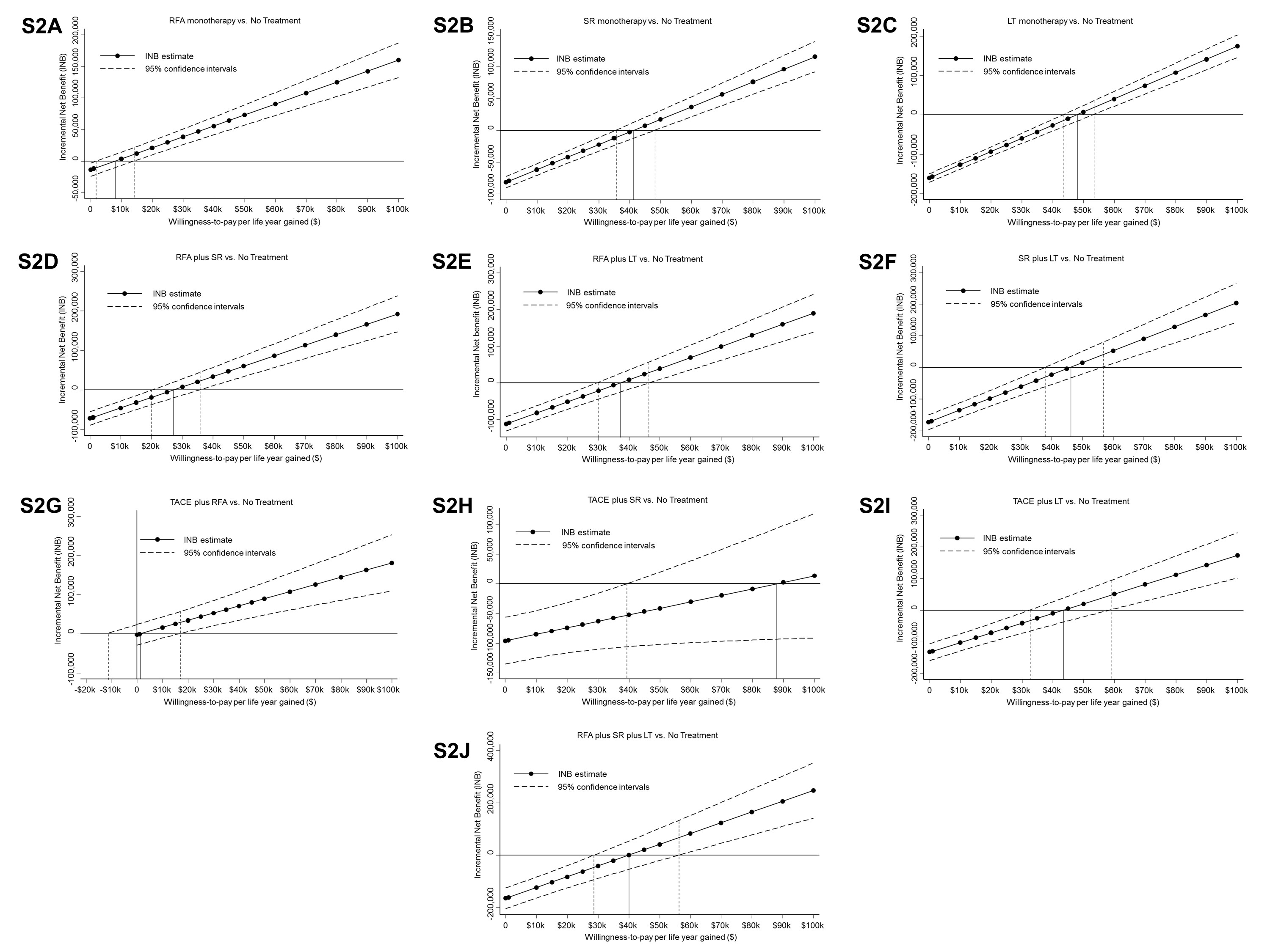

Supplement: Supplementary file 2 — Figure S2. Estimates of incremental net benefit (i.e., incremental cost‐effectiveness ratio, ICER) and its 95% confidence intervals as a function of willingness‐to‐pay threshold for an additional life year (LY); (S2A) radiofrequency ablation (RFA) monotherapy versus no treatment; (S2B) surgical resection (SR) monotherapy versus no treatment; (S2C) liver transplantation (LT) monotherapy versus no treatment; (S2D) RFA plus SR versus no treatment; (S2E) RFA plus LT versus no treatment; (S2F) SR plus LT versus no treatment; (S2G) TACE plus RFA versus no treatment; (S2H) TACE plus SR versus no treatment; (S2I) TACE plus LT versus no treatment; and (S2J) RFA plus SR plus LT versus no treatment. [file CAM4-6-2017-s002.tif]

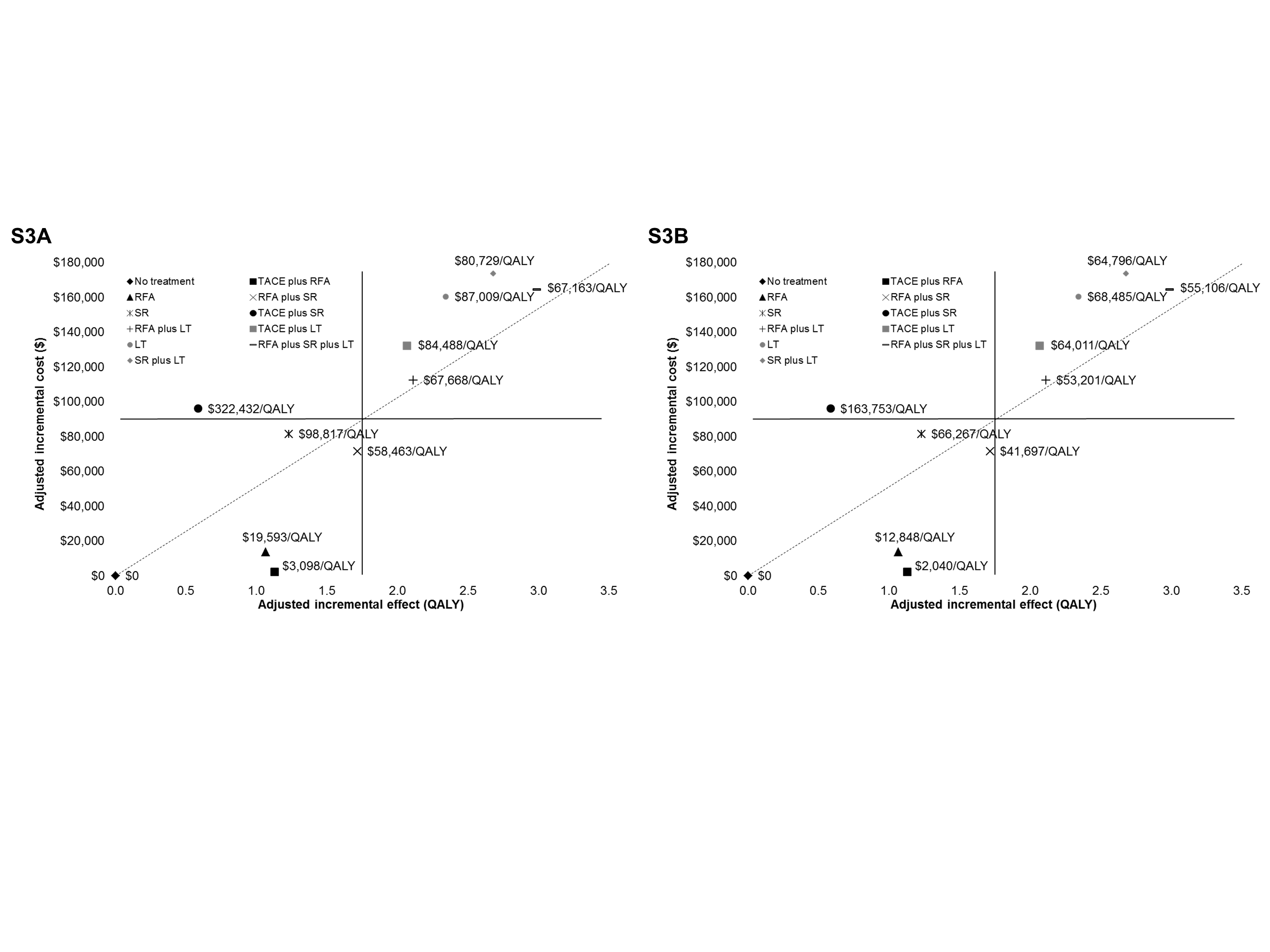

Supplement: Supplementary file 3 — Figure S3. Efficiency frontier: plot of incremental quality‐adjusted life years (QALYs) and costs of curative treatments relative to lowest cost scenario (no treatment): Sensitivity analysis according to (S3A) lower bound and (S3B) upper bound of pooled mean health state utilities of disease stage from published literature. The dotted diagonal line represents the willingness‐to‐pay for health effects (maximum acceptable ceiling ratio). If an intervention lies above the line, it will not be acceptable on cost‐effectiveness grounds. [file CAM4-6-2017-s003.tif]

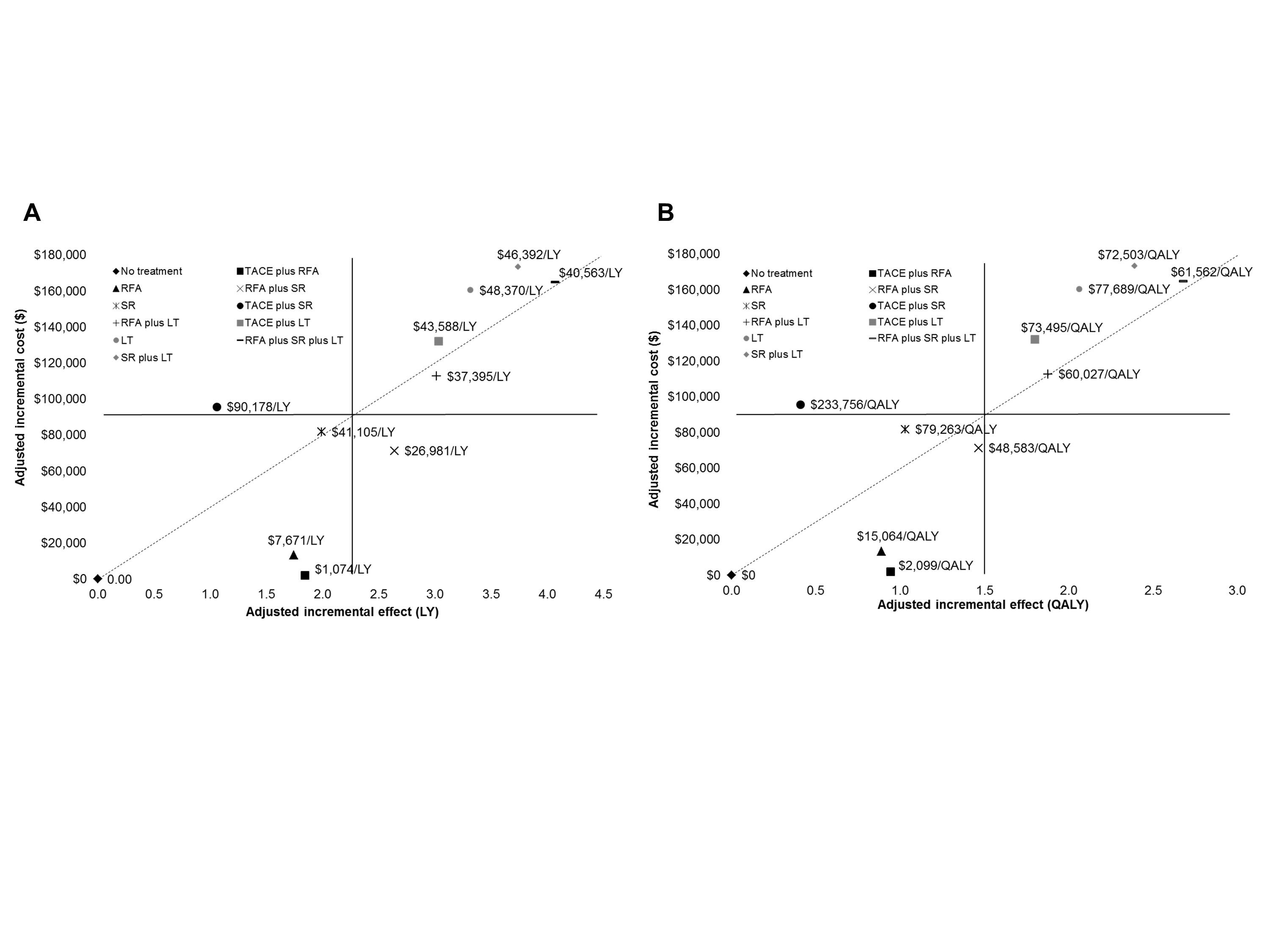

Supplement: Supplementary file 4 — Figure S4. Efficiency frontier: Efficiency frontier: plot of incremental (A) life years (LYs) and (B) quality‐adjusted life years (QALYs) and costs of curative treatments relative to lowest cost scenario (no treatment): Sensitivity analysis of excluding HCC stage IV. [file CAM4-6-2017-s004.tif]
