# Supplementary material for: Cost‐effectiveness analysis of potentially curative and combination treatments for hepatocellular carcinoma with person‐level data in a Canadian setting
Source: Cancer Med. 2017 Aug 8;6(9):2017–33. doi: 10.1002/cam4.1119 (PMC5603843; doi:10.1002/cam4.1119)
Supplement: Supplementary file 5 — Tables S1. ICD‐9 and ICD‐10 codes for patients diagnosed with diabetes, HIV, and liver disease stage. Table S2. Screening and treatment procedures for patients with hepatocellular carcinoma. Table S3. Estimation of utilities for noncirrhosis. Table S4. Baseline characteristics of patients with hepatocellular carcinoma, 2002–2010. Table S5. Estimates of incremental net benefit and probability of cost‐effectiveness of curative treatment strategies for hepatocellular carcinoma compared with no treatment as a function of willingness‐to‐pay threshold per additional life year over the study period 2002–2010. [file CAM4-6-2017-s005.docx]

**Supporting Information**

Full details of methodological descriptions regarding data sources and estimation of direct healthcare costs associated with hepatocellular carcinoma (HCC) can be found elsewhere.^1-4^ HCC cases were identified only using ICD-9 coding due to the lack of ICD-10 C22 code in the dataset.

Supporting Table 1. ICD-9 and ICD-10 codes for patients diagnosed with diabetes, HIV, and liver disease stage

|  | ICD-9 code | ICD-10 code |
| --- | --- | --- |
| Diabetes | 250 | E10-E14 |
| HIV | 042, 043, 044 | B20-B24 |
| Cirrhosis | 571 | K74 |
| Alcoholic liver disease | 571.0, 571.1, 571.2, 571.3 | K70 |
| Non-alcoholic liver disease | 571.8 | K76.0 |
| Ascites | 789.5 | R18 |
| Esophageal varices | 456.0, 456.1, 456.2 | I85 |
| Hepatic encephalopathy | 572.2 | K72 |

ICD-9, the International Classification of Diseases, 9th revision; ICD-10, International Statistical Classification of Diseases and Related Health Problems, 10th revision.

Supporting Table 2. Screening and treatment procedures for patients with hepatocellular carcinoma

|  | Code type^*^ | | |
| --- | --- | --- | --- |
|  | CCP | CCI | OHIP |
| **Screening** |  |  |  |
| Diagnostic ultrasound-abdomen-abdominal scan-limited study |  |  | J128 |
| Diagnostic ultrasound-abdomen/retroperitoneal abdominal scan complete |  |  | J135 |
| Diagnostic ultrasound-abdomen & retroperitoneal.p2-abdominal scan-limited study |  |  | J428 |
| Diagnostic ultrasound- abdomen & retroperitoneal.p2-abdominal scan complete |  |  | J435 |
|  |  |  |  |
| **Treatment** |  |  |  |
| ***Potentially curative therapy*** |  |  |  |
| Local excision or destruction of lesion or tissue of liver | 62.1 |  |  |
| Partial hepatectomy | 62.12 |  |  |
| Other destruction of lesion of liver | 62.19 |  |  |
| Lobectomy of liver | 62.20 |  |  |
| Excision partial, liver, using endoscopic (laparoscopic) approach |  | 1OA87DA |  |
| Excision partial, liver, using open approach |  | 1OA87LA |  |
| Excision partial, liver, using ultrasonic aspirator device (for dissection) and open approach |  | 1OA87LAAZ |  |
| Liver excision, complete left or right lobectomy |  |  | S267 |
| Liver excision of lesion |  |  | S269 |
| Liver excision, hepatectomy, left lateral segmental excision |  |  | S270 |
| Liver excision, extended right lobectomy |  |  | S271 |
| Liver excision, partial lobectomy |  |  | S275 |
| Total hepatectomy | 62.3 |  |  |
| Liver transplant | 62.4 |  |  |
| Auxiliary liver transplant | 62.41 |  |  |
| Other transplant of liver | 62.49 |  |  |
| Transplant, liver of a deceased donor full size liver |  | 1OA85LAXXK |  |
| Transplant, liver of a deceased donor, multiorgan liver with intestine, pancreas, spleen, or stomach (or any combination of) |  | 1OA85VCXXK |  |
| Transplant, liver of a living donor, split liver |  | 1OA85WLXXJ |  |
| Transplant, liver of a deceased donor split liver (or reduced paediatric-size liver) |  | 1OA85WLXXK |  |
| Living donor orthotopic liver transplantation recipient |  |  | S266 |
| Liver excision, liver transplant recipient |  |  | S294 |
| Digestive system-liver, repeat liver transplant |  |  | S295 |
| Destruction, liver endoscopic (laparoscopic) approach using radiofrequency |  | 1OA59DAAW |  |
| Destruction, liver percutaneous approach using radiofrequency |  | 1OA59HAAW |  |
| Destruction, liver open approach using radiofrequency |  | 1OA59LAAW |  |
| Radiofrequency ablation |  |  | J069 |

Supporting Table 2. Screening and treatment procedures for patients with hepatocellular carcinoma (continued)

|  | Code type^*^ | | |
| --- | --- | --- | --- |
|  | CCP | CCI | OHIP |
| ***Non-curative therapy*** |  |  |  |
| Percutaneous ablation |  |  |  |
| Destruction, liver endoscopic (abdominal) approach using cryoprobe |  | 1OA59DAAD |  |
| Destruction, liver endoscopic (abdominal) approach using laser |  | 1OA59DAAG |  |
| Destruction, liver endoscopic (abdominal) approach using device NEC |  | 1OA59DAGX |  |
| Destruction, liver endoscopic (abdominal) approach using chemical cautery agent (e.g. ethanol) |  | 1OA59DAX7 |  |
| Destruction, liver percutaneous approach using chemical cautery agent (e.g. ethanol) |  | 1OA59HAX7 |  |
| Destruction, liver open approach using cryoprobe |  | 1OA59LAAD |  |
| Destruction, liver open approach using laser |  | 1OA59LAAG |  |
| Destruction, liver open approach using device NEC |  | 1OA59LAGX |  |
| Destruction, liver open approach using chemical cautery agent (e.g. ethanol) |  | 1OA59LAX7 |  |
| Chemotherapy |  |  |  |
| Diagnostic and therapeutic injection(s) or infusion(s), test dose (bleomycin and L-asparatiginase once per patient per drug) |  |  | G075 |
| Diagnostic and therapeutic injection or infusion (intravenous chemotherapy), each additional injection |  |  | G281 |
| Single-agent intravenous chemotherapy, i.e. doxorubicin, daunorubicin, epirubicin, mitoxintrone, cisplatin or bleomycin (>10 U/m^2^) |  |  | G339 |
| Taxol, rituximab, trastuzumab, bortezomib, docetaxel administration or multiple agent intravenous chemotherapy including at least one of either doxorubicin, daunorubicin, epirubicin, mitoxintrone, cisplatin or bleomycin (>10 U/m^2^) |  |  | G345 |
| Special single agent chemotherapy utilizing either high-dose methotrexate with folinic acid rescue - methotrexate given in a dose of >1 g/m^2^, high dose cisplatin >75 mg/m^2^ given concurrently with hydration and osmotic diuresis, high dose cystosine, arabinoside (>2g/m^2^), or high dose cyclophosphamide (>1g/m^2^) |  |  | G359 |
| Single injection (for agents other than doxorubicin, cisplatin,  bleomycin or high dose methotrexate) |  |  | G381 |
| Supervision of chemotherapy (marrow suppressant) for  malignant or autoimmune disease by telephone - monthly |  |  | G382 |
| Arteries-cannulation-chemotherapy-hepatic (TACE) |  |  | R776 |
| ***Supportive/Palliative care*** |  |  |  |
| General or family practice, special palliative care consultation |  |  | A945 |
| Special palliative care consultation, hospital inpatient |  |  | C945 |
| Palliative care |  |  | C982 |
| Palliative care support individual care, 0.5 hours or major part |  |  | K023 |

*The CCI is the new national standard for classifying health care procedures. It is the companion classification system to the International Statistical Classification of Diseases and Related Health Problems, 10th revision, Canada, and replaces the CCP and the intervention portion of the International Classification of Diseases, 9th revision, Clinical Modification, in Canada. The CCP was originally developed by Statistics Canada in 1978 to meet Canadian needs for a procedural classification to be used in conjunction with the International Classification of Diseases, 9th revision.

CCP, Canadian Classification of Diagnostic, Therapeutic and Surgical Procedures; CCI, Canadian Classification of Health Interventions; OHIP, Ontario Health Insurance Plan; NEC = not elsewhere classified; TACE, transarterial chemoembolization.

Supporting Table 3A. Estimation of utilities for non-cirrhosis

| Author, year | Preference-based measures | Country | Mean | Standard error | Lower limit | Upper limit |
| --- | --- | --- | --- | --- | --- | --- |
| Chong et al, 2003^5^ | EQ-5D | Canada | 0.76 | 0.041 | 0.67964 | 0.84036 |
| Chong et al, 2003^5^ | HUI3 | Canada | 0.73 | 0.05 | 0.632 | 0.828 |
| Chong et al, 2003^5^ | SG | Canada | 0.79 | 0.04 | 0.7116 | 0.8684 |
| Sherman et al, 2004^6^ | SG | US | 0.81 | 0.04 | 0.7316 | 0.8884 |
| Sherman et al, 2004^6^ | TTO | US | 0.85 | 0.04 | 0.7716 | 0.9284 |
| Siebert et al, 2001^7,8^ | EQ-5D | Germany | 0.76 | 0.02 | 0.7208 | 0.7992 |
| Younossi et al, 2001^9^ | HUI2 | US | 0.84 | 0.03 | 0.7812 | 0.8988 |
| Wright et al, 2006^10^ | EQ-5D | UK | 0.66 | 0.03 | 0.6012 | 0.7188 |
| Hsu et al, 2012^11^ | HUI2 | Canada | 0.73 | 0.012 | 0.70648 | 0.75352 |
| Hsu et al, 2012^11^ | SF-6D | Canada | 0.66 | 0.008 | 0.64432 | 0.67568 |
| Hsu et al, 2012^11^ | TTO | Canada | 0.8 | 0.014 | 0.77256 | 0.82744 |
| Fixed |  |  | 0.718 | 0.005 | 0.707 | 0.728 |
| Random |  |  | 0.760 | 0.023 | 0.716 | 0.805 |

Assessment of heterogeneity: *I*^2^ = 92.5%; *P*<0.001.

EQ, EuroQoL; HUI, Health Utilities Index; SG, Standard Gamble; TTO, Time Trade-Off; SF, Short Form Health Survey.

Supporting Table 3B. Estimation of utilities for compensated cirrhosis

| Author, year | Preference-based measures | Country | Mean | Standard error | Lower limit | Upper limit |
| --- | --- | --- | --- | --- | --- | --- |
| Chong et al, 2003^5^ | EQ-5D | Canada | 0.74 | 0.05 | 0.642 | 0.838 |
| Chong et al, 2003^5^ | HUI3 | Canada | 0.74 | 0.05 | 0.642 | 0.838 |
| Chong et al, 2003^5^ | SG | Canada | 0.8 | 0.05 | 0.702 | 0.898 |
| Sherman et al, 2004^6^ | SG | US | 0.83 | 0.04 | 0.7516 | 0.9084 |
| Sherman et al, 2004^6^ | TTO | US | 0.9 | 0.03 | 0.8412 | 0.9588 |
| Siebert et al, 2001^7,8^ | EQ-5D | Germany | 0.74 | 0.02 | 0.7008 | 0.7792 |
| Younossi et al, 2001^9^ | HUI2 | US | 0.82 | 0.04 | 0.7416 | 0.8984 |
| Hsu et al, 2012^11^ | HUI2 | Canada | 0.73 | 0.012 | 0.70648 | 0.75352 |
| Hsu et al, 2012^11^ | SF-6D | Canada | 0.66 | 0.008 | 0.64432 | 0.67568 |
| Hsu et al, 2012^11^ | TTO | Canada | 0.78 | 0.021 | 0.73884 | 0.82116 |
| Fixed |  |  | 0.710 | 0.006 | 0.699 | 0.721 |
| Random |  |  | 0.771 | 0.025 | 0.722 | 0.820 |

Assessment of heterogeneity: *I*^2^ = 92.2%; *P*<0.001.

Supporting Table 3C. Estimation of utilities for decompensated cirrhosis

| Author, year | Preference-based measures | Country | Mean | Standard error | Lower limit | Upper limit |
| --- | --- | --- | --- | --- | --- | --- |
| Chong et al, 2003^5^ | EQ-5D | Canada | 0.66 | 0.1 | 0.464 | 0.856 |
| Chong et al, 2003^5^ | HUI3 | Canada | 0.69 | 0.08 | 0.5332 | 0.8468 |
| Chong et al, 2003^5^ | SG | Canada | 0.6 | 0.12 | 0.3648 | 0.8352 |
| Sherman et al, 2004^6^ | SG | US | 0.72 | 0.12 | 0.4848 | 0.9552 |
| Sherman et al, 2004^6^ | TTO | US | 0.72 | 0.12 | 0.4848 | 0.9552 |
| Siebert et al, 2001^7,8^ | EQ-5D | Germany | 0.72 | 0.03 | 0.6612 | 0.7788 |
| Younossi et al, 2001^9^ | HUI2 | US | 0.71 | 0.1 | 0.514 | 0.906 |
| Fixed |  |  | 0.708 | 0.024 | 0.660 | 0.756 |
| Random |  |  | 0.708 | 0.024 | 0.660 | 0.756 |

Assessment of heterogeneity: *I*^2^ = 0%; *P*=0.973.

Supporting Table 3D. Estimation of utilities for post liver transplantation

| Author, year | Preference-based measures | Country | Mean | Standard error | Lower limit | Upper limit |
| --- | --- | --- | --- | --- | --- | --- |
| Chong et al, 2003^5^ | EQ-5D | Canada | 0.69 | 0.04 | 0.6116 | 0.7684 |
| Chong et al, 2003^5^ | HUI3 | Canada | 0.7 | 0.04 | 0.6216 | 0.7784 |
| Chong et al, 2003^5^ | SG | Canada | 0.73 | 0.06 | 0.6124 | 0.8476 |
| Sherman et al, 2004^6^ | SG | US | 0.72 | 0.1 | 0.524 | 0.916 |
| Sherman et al, 2004^6^ | TTO | US | 0.81 | 0.1 | 0.614 | 1.006 |
| Hsu et al, 2012^11^ | HUI2 | Canada | 0.75 | 0.024 | 0.70296 | 0.79704 |
| Hsu et al, 2012^11^ | SF-6D | Canada | 0.65 | 0.016 | 0.61864 | 0.68136 |
| Hsu et al, 2012^11^ | TTO | Canada | 0.8 | 0.038 | 0.72552 | 0.87448 |
| Fixed |  |  | 0.697 | 0.011 | 0.675 | 0.719 |
| Random |  |  | 0.721 | 0.025 | 0.673 | 0.770 |

Assessment of heterogeneity: *I*^2^ = 68.9%; *P*=0.002.

Description of the net benefit regression model

To examine this, we employed an empirical model that interacts ten treatment dummy variables with the covariates as follows:

$$NB({\lambda)}_{i} = +\sum_{j=1}^{n} \beta_{j}x_{ij}+ \delta1{T1}_{i \ldots\ldots.}+ \delta10{T10}_{i}+\sum_{j=1}^{n} \gamma_{j}{T_{i}x}_{ij}+ \varepsilon_{i}$$

where: NB(λ)_i_ is the person-level NB; α is an intercept term; T_i_ is a treatment dummy, indicating whether person i received treatment (i.e. T_i_=1) or no treatment (i.e. T_i_=0); β is the coefficient estimate for the aforementioned covariates of interest x; γ is an interaction term between subject characteristic and the treatment indicator; and ε is a stochastic error term assumed to be normally distributed.

The final net benefit model comprised of:

${NB}_{i}$ = α + δ_1_(*T_1_*)_i_ ……+ δ_10_(*T_10_*)_i_ + β_1_(age)_i_ + β_2_(sex)_i_ + β_3_(income quintile)_i_ + β_4_(urban or rural residence)_i_ + β_5_(birth country)_i_ + β_6_(Charlson-Deyo comorbidity index)_i_ + β_7_(diabetes)_i_ + β_8_(HIV)_i_ + β_9_(indicators of liver disease stage)_i_ + β_10_(ultrasound screening)_i_ + β_11_(stage at HCC diagnosis)_i_ + β_12_(index year)_i_ + β_13_(propensity score)_i_ + ε_i_

Supporting Table 4. Baseline characteristics of patients with hepatocellular carcinoma, 2002-2010

| **Variable** | **N (%)** |
| --- | --- |
| Overall | 2222 (100%) |
| Age group (years) |  |
| <60 | 808 (36.4) |
| 60-69 | 586 (26.4) |
| 70-79 | 596 (26.8) |
| 80+ | 232 (10.4) |
| Sex |  |
| Female | 486 (21.9) |
| Male | 1736 (78.1) |
| Income quintile |  |
| Q1 (lowest) | 539 (24.3) |
| Q2 | 479 (21.6) |
| Q3 | 456 (20.5) |
| Q4 | 381 (17.2) |
| Q5 (highest) | 354 (15.9) |
| Missing | 13 (0.6) |
| Residence |  |
| Urban | 2030 (91.4) |
| Rural | 189 (8.5) |
| Missing | - (0.1) |
| Birth country |  |
| Other | 743 (33.4) |
| Canada | 728 (32.8) |
| Unknown/Missing | 751 (33.8) |
| Charlson-Deyo comorbidity index |  |
| 0 | 808 (36.4) |
| 1 | 637 (28.7) |
| 2 | 271 (12.2) |
| 3+ | 192 (8.6) |
| No hospitalization record | 314 (14.1) |
| Diabetes diagnosis | 1114 (50.1) |
| HIV | 50 (2.3) |

Supporting Table 4 continued on the following page

Supporting Table 4. Total characteristics of patients with hepatocellular carcinoma, 2002-2010 (continued)

| **Variable** | **N (%)** |
| --- | --- |
| Indicators of liver disease stage |  |
| Viral hepatitis only | 48 (2.2) |
| No cirrhosis | 412 (18.5) |
| Cirrhosis only | 426 (19.2) |
| ALD + Cirrhosis | 70 (3.2) |
| Viral hepatitis + Cirrhosis | 78 (3.5) |
| ALD + Viral hepatitis + Cirrhosis | 15 (0.7) |
| Decompensated cirrhosis only | 594 (26.7) |
| ALD + Decompensated cirrhosis | 308 (13.9) |
| NAFLD + Decompensated cirrhosis | 21 (1.0) |
| Viral hepatitis + Decompensated cirrhosis | 115 (5.2) |
| ALD + Viral Hepatitis + Decompensated cirrhosis | 78 (3.5) |
| Ultrasound screening 2 years before HCC diagnosis |  |
| No screening | 1034 (46.5) |
| Inconsistent screening | 926 (41.7) |
| ≥1 screens annually | 262 (11.8) |
| Stage at HCC diagnosis |  |
| Early (stage I) | 289 (13.0) |
| Intermediate (stage II) | 315 (14.2) |
| Advanced (stage III-IV) | 370 (16.7) |
| Unknown | 1248 (56.2) |
| Year of HCC diagnosis |  |
| 2002 | 196 (8.8) |
| 2003 | 188 (8.5) |
| 2004 | 221 (10.0) |
| 2005 | 247 (11.1) |
| 2006 | 259 (11.7) |
| 2007 | 260 (11.7) |
| 2008 | 247 (11.1) |
| 2009 | 304 (13.7) |
| 2010 | 300 (13.5) |

Supporting Table 5. Estimates of incremental net benefit and probability of cost effectiveness of curative treatment strategies for hepatocellular carcinoma compared with no treatment as a function of willingness-to-pay threshold per additional life year over the study period 2002-2010

| λ thresholds | Radiofrequency Ablation | | |  | Surgical Resection | | |  | Liver Transplantation | | |
| --- | --- | --- | --- | --- | --- | --- | --- | --- | --- | --- | --- |
|  | INB estimate (SE) | *P*-value^*^ | Probability of cost-effectiveness |  | INB estimate (SE) | *P*-value^*^ | Probability of cost-effectiveness |  | INB estimate (SE) | *P*-value^*^ | Probability of cost-effectiveness |
| $0 | -13698 (5332) | 0.005 | 0.0051 |  | -81536 (4592) | <0.001 | 0.0001 |  | -160428 (5568) | <0.001 | 0.0001 |
| $1,000 | -11968 (5316) | 0.012 | 0.0122 |  | -79563 (4580) | <0.001 | 0.0001 |  | -157080 (5551) | <0.001 | 0.0001 |
| $10,000 | 3599 (5327) | 0.250 | 0.7504 |  | -61804 (4608) | <0.001 | 0.0001 |  | -126952 (5566) | <0.001 | 0.0001 |
| $20,000 | 20896 (5666) | <0.001 | 0.9999 |  | -42072 (4916) | <0.001 | 0.0001 |  | -93476 (5924) | <0.001 | 0.0001 |
| $30,000 | 38193 (6291) | <0.001 | 1.0000 |  | -22340 (5468) | <0.001 | 0.0001 |  | -60001 (6584) | <0.001 | 0.0001 |
| $40,000 | 55490 (7129) | <0.001 | 1.0000 |  | -2608 (6198) | 0.337 | 0.3370 |  | -26525 (7466) | <0.001 | 0.0002 |
| $50,000 | 72787 (8114) | <0.001 | 1.0000 |  | 17124 (7052) | 0.008 | 0.9924 |  | 6951 (8501) | 0.207 | 0.7933 |
| $60,000 | 90084 (9199) | <0.001 | 1.0000 |  | 36856 (7989) | <0.001 | 1.0000 |  | 40427 (9640) | <0.001 | 1.0000 |
| $70,000 | 107380 (10352) | <0.001 | 1.0000 |  | 56588 (8984) | <0.001 | 1.0000 |  | 73903 (10850) | <0.001 | 1.0000 |
| $80,000 | 124677 (11553) | <0.001 | 1.0000 |  | 76320 (10019) | <0.001 | 1.0000 |  | 107378 (12111) | <0.001 | 1.0000 |
| $90,000 | 141974 (12789) | <0.001 | 1.0000 |  | 96052 (11084) | <0.001 | 1.0000 |  | 140854 (13407) | <0.001 | 1.0000 |
| $100,000 | 159271 (14050) | <0.001 | 1.0000 |  | 115784 (12170) | <0.001 | 1.0000 |  | 174330 (14730) | <0.001 | 1.0000 |

Supporting Table 5 continued on the following page

Supporting Table 5. Estimates of incremental net benefit and probability of cost effectiveness of curative treatment strategies for hepatocellular carcinoma compared with no treatment as a function of willingness-to-pay threshold per additional life year over the study period 2002-2010 (continued)

| λ thresholds | Radiofrequency Ablation plus Surgical Resection | | |  | Radiofrequency Ablation plus Liver Transplantation | | |  | Surgical Resection plus Liver Transplantation | | |
| --- | --- | --- | --- | --- | --- | --- | --- | --- | --- | --- | --- |
|  | INB estimate (SE) | *P*-value^*^ | Probability of cost-effectiveness |  | INB estimate (SE) | *P*-value^*^ | Probability of cost-effectiveness |  | INB estimate (SE) | *P*-value^*^ | Probability of cost-effectiveness |
| $0 | -71514 (8749) | <0.001 | 0.0001 |  | -112421 (9864) | <0.001 | 0.0001 |  | -173510 (11812) | <0.001 | 0.0001 |
| $1,000 | -68877 (8726) | <0.001 | 0.0001 |  | -109403 (9838) | <0.001 | 0.0001 |  | -169751 (11783) | <0.001 | 0.0001 |
| $10,000 | -45139 (8781) | <0.001 | 0.0001 |  | -82240 (9899) | <0.001 | 0.0001 |  | -135922 (11877) | <0.001 | 0.0001 |
| $20,000 | -18764 (9373) | 0.023 | 0.0227 |  | -52059 (10568) | <0.001 | 0.0001 |  | -98334 (12690) | <0.001 | 0.0001 |
| $30,000 | 7611 (10429) | 0.233 | 0.7673 |  | -21879 (11762) | 0.031 | 0.0315 |  | -60746 (14123) | <0.001 | 0.0001 |
| $40,000 | 33986 (11826) | 0.002 | 0.9980 |  | 8302 (13342) | 0.267 | 0.7331 |  | -23158 (16011) | 0.074 | 0.0741 |
| $50,000 | 60362 (13458) | <0.001 | 1.0000 |  | 38483 (15188) | 0.006 | 0.9944 |  | 14430 (18212) | 0.214 | 0.7859 |
| $60,000 | 86737 (15250) | <0.001 | 1.0000 |  | 68664 (17214) | <0.001 | 1.0000 |  | 52018 (20626) | 0.006 | 0.9942 |
| $70,000 | 113112 (17151) | <0.001 | 1.0000 |  | 98844 (19363) | <0.001 | 1.0000 |  | 89606 (23188) | <0.001 | 1.0000 |
| $80,000 | 139487 (19130) | <0.001 | 1.0000 |  | 129025 (21600) | <0.001 | 1.0000 |  | 127194 (25852) | <0.001 | 1.0000 |
| $90,000 | 165862 (21164) | <0.001 | 1.0000 |  | 159206 (23899) | <0.001 | 1.0000 |  | 164782 (28591) | <0.001 | 1.0000 |
| $100,000 | 192238 (23239) | <0.001 | 1.0000 |  | 189387 (26245) | <0.001 | 1.0000 |  | 202370 (31385) | <0.001 | 1.0000 |

Supporting Table 5 continued on the following page

Supporting Table 5. Estimates of incremental net benefit and probability of cost effectiveness of curative treatment strategies for hepatocellular carcinoma compared with no treatment as a function of willingness-to-pay threshold per additional life year over the study period 2002-2010 (continued)

| λ thresholds | TACE plus Radiofrequency Ablation | | |  | TACE plus Surgical Resection | | |  | TACE plus Liver Transplantation | | |
| --- | --- | --- | --- | --- | --- | --- | --- | --- | --- | --- | --- |
|  | INB estimate (SE) | *P*-value^*^ | Probability of cost-effectiveness |  | INB estimate (SE) | *P*-value^*^ | Probability of cost-effectiveness |  | INB estimate (SE) | *P*-value^*^ | Probability of cost-effectiveness |
| $0 | -2262 (13639) | 0.434 | 0.4342 |  | -95930 (20116) | <0.001 | 0.0001 |  | -132378 (13697) | <0.001 | 0.0001 |
| $1,000 | -434 (13604) | 0.487 | 0.4873 |  | -94837 (20067) | <0.001 | 0.0001 |  | -129327 (13662) | <0.001 | 0.0001 |
| $10,000 | 16020 (13700) | 0.121 | 0.8789 |  | -85000 (20229) | <0.001 | 0.0001 |  | -101871 (13761) | <0.001 | 0.0001 |
| $20,000 | 34302 (14634) | 0.010 | 0.9905 |  | -74069 (21614) | <0.001 | 0.0003 |  | -71363 (14700) | <0.001 | 0.0001 |
| $30,000 | 52585 (16293) | 0.001 | 0.9994 |  | -63139 (24052) | 0.004 | 0.0044 |  | -40856 (16366) | 0.006 | 0.0063 |
| $40,000 | 70867 (18483) | <0.001 | 1.0000 |  | -52208 (27262) | 0.028 | 0.0278 |  | -10349 (18562) | 0.289 | 0.2886 |
| $50,000 | 89149 (21037) | <0.001 | 1.0000 |  | -41277 (31005) | 0.092 | 0.0916 |  | 20158 (21124) | 0.170 | 0.8300 |
| $60,000 | 107431 (23840) | <0.001 | 1.0000 |  | -30347 (35112) | 0.194 | 0.1937 |  | 50665 (23936) | 0.017 | 0.9829 |
| $70,000 | 125713 (26814) | <0.001 | 1.0000 |  | -19416 (39468) | 0.311 | 0.3114 |  | 81173 (26918) | 0.001 | 0.9987 |
| $80,000 | 143995 (29907) | <0.001 | 1.0000 |  | -8486 (44000) | 0.424 | 0.4236 |  | 111680 (30021) | <0.001 | 0.9999 |
| $90,000 | 162277 (33086) | <0.001 | 1.0000 |  | 2445 (48659) | 0.480 | 0.5201 |  | 142187 (33210) | <0.001 | 1.0000 |
| $100,000 | 180559 (36329) | <0.001 | 1.0000 |  | 13376 (53411) | 0.401 | 0.5989 |  | 172694 (36463) | <0.001 | 1.0000 |

Supporting Table 5 continued on the following page

Supporting Table 5. Estimates of incremental net benefit and probability of cost effectiveness of curative treatment strategies for hepatocellular carcinoma compared with no treatment as a function of willingness-to-pay threshold per additional life year over the study period 2002-2010 (continued)

| λ thresholds | Radiofrequency Ablation plus Surgical Resection plus Liver Transplantation | | |
| --- | --- | --- | --- |
|  | INB estimate (SE) | *P*-value^*^ | Probability of cost-effectiveness |
| $0 | -164675 (20178) | <0.001 | 0.0001 |
| $1,000 | -160565 (20129) | <0.001 | 0.0001 |
| $10,000 | -123581 (20294) | <0.001 | 0.0001 |
| $20,000 | -82488 (21691) | <0.001 | 0.0001 |
| $30,000 | -41394 (24147) | 0.043 | 0.0433 |
| $40,000 | -300 (27379) | 0.496 | 0.4957 |
| $50,000 | 40793 (31146) | 0.095 | 0.9049 |
| $60,000 | 81887 (35278) | 0.010 | 0.9899 |
| $70,000 | 122981 (39660) | 0.001 | 0.9991 |
| $80,000 | 164074 (44218) | <0.001 | 0.9999 |
| $90,000 | 205168 (48903) | <0.001 | 1.0000 |
| $100,000 | 246262 (53682) | <0.001 | 1.0000 |

^*^one-sided *P*-value. λ, willingness-to-pay; INB, incremental net benefit; SE, standard error.

**References**

1. Jembere N, Campitelli MA, Sherman M*, et al.* Influence of socioeconomic status on survival of hepatocellular carcinoma in the Ontario population; a population-based study, 1990-2009. *PLoS One* 2012;7:e40917.

2. Thein HH, Isaranuwatchai W, Campitelli MA*, et al.* Health care costs associated with hepatocellular carcinoma: a population-based study. *Hepatology* 2013;58:1375-84.

3. Thein HH, Campitelli MA, Yeung LT, Zaheen A, Yoshida EM, Earle CC. Improved Survival in Patients with Viral Hepatitis-Induced Hepatocellular Carcinoma Undergoing Recommended Abdominal Ultrasound Surveillance in Ontario: A Population-Based Retrospective Cohort Study. *PLoS One* 2015;10:e0138907.

4. Thein HH, Qiao Y, Young SK*, et al.* Trends in health care utilization and costs attributable to hepatocellular carcinoma, 2002-2009: a population-based cohort study. *Curr Oncol* 2016;23:e196-220.

5. Chong CA, Gulamhussein A, Heathcote EJ*, et al.* Health-state utilities and quality of life in hepatitis C patients. *Am J Gastroenterol* 2003;98:630-8.

6. Sherman KE, Sherman SN, Chenier T, Tsevat J. Health values of patients with chronic hepatitis C infection. *Arch Intern Med* 2004;164:2377-82.

7. Siebert U, Sieberer R, Greiner W, et al. Patient-based health-related quality of life in different stages of chronic hepatitis C [Abstract]. Hepatology 2001;34(Pt 2): AB222A.

8. McLernon DJ, Dillon J, Donnan PT. Health-state utilities in liver disease: a systematic review. *Med Decis Making* 2008;28:582-92.

9. Younossi ZM, Boparai N, McCormick M, Price LL, Guyatt G. Assessment of utilities and health-related quality of life in patients with chronic liver disease. *Am J Gastroenterol* 2001;96:579-83.

10. Wright M, Grieve R, Roberts J, Main J, Thomas HC. Health benefits of antiviral therapy for mild chronic hepatitis C: randomised controlled trial and economic evaluation. *Health Technol Assess* 2006;10:1-113, iii.

11. Hsu PC, Federico CA, Krajden M*, et al.* Health utilities and psychometric quality of life in patients with early- and late-stage hepatitis C virus infection. *J Gastroenterol Hepatol* 2012;27:149-57.
